# TrkC-mediated inhibition of DJ-1 degradation is essential for direct regulation of pathogenesis of hepatocellular carcinoma

**DOI:** 10.1038/s41419-022-05298-3

**Published:** 2022-10-06

**Authors:** Min Soo Kim, Won Sung Lee, Yeonmi Park, Wook Jin

**Affiliations:** 1 Laboratory of Molecular Disease and Cell Regulation, Department of Biochemistry, School of Medicine, Gachon University, Incheon, 21999 Republic of Korea; 2 Department of Health Sciences and Technology, GAIHST, Gachon University, Incheon, 21999 Korea

**Keywords:** Metastasis, Cell invasion

## Abstract

None of the previous studies has systematically explored how upregulation of TrkC plays a central role in the pathogenesis of hepatocellular carcinoma (HCC) by regulating the underlying mechanisms that promote invasion and metastasis. In this report, we demonstrated the possible association between upregulation of TrkC and acquisition of cancer stem cells traits or chemoresistance in HCC. We show that upregulation of TrkC is closely associated with the survival and progression of HCC in vivo and in vitro. Most strikingly, activation of STAT3 by TrkC-mediated inhibition of DJ-1 degradation significantly enhances the efficacy of invasion and metastasis during the progression of HCC cells. Acquiring the traits of cancer stem cells (CSCs) by TrkC/DJ-1/STAT3 signaling pathway leads to the induction of chemoresistance via upregulation of ABC transporters and anti-apoptotic genes. Also, activating the epithelial-mesenchymal transition (EMT) program by inducing EMT-transcription factor (TF)s by TrkC/DJ-1/STAT3 signaling pathway is the direct cause of multiple tumor malignancies of HCC. Thus, understanding the mechanisms by which acquisition of anticancer drug resistance by TrkC-mediated inhibition of DJ-1 degradation can help enhance the efficacy of anticancer therapies.

## Introduction

The activation of the epithelial-mesenchymal transition (EMT) program plays a critical role in acquiring the high-grade malignant traits of cancer cells through interconverting from epithelial to mesenchymal phenotypes. Thus, induction of the EMT program is closely associated with highly metastatic cancer subtypes and poor survival of cancer patients [1–3]. In this response, diverse cellular signalings, including transforming growth factor (TGF)-β, canonical and noncanonical Wnt signaling, and the expression of EMT core signature by various EMT transcription factors (EMT-TFs), such as Snail, Slug, SIP-1, Goosecoid, Foxc1, Foxc2, Twist-1, and Twist-2, collaborate to induce the EMT program [3, 4]. In addition, activation of the EMT program via upregulation of EMT-TFs by interacting between EMT-TFs as a network exhibited the direct mechanistic link to the acquisition of cancer stem cells and anticancer drug resistance. Induction of EMT generates cells with properties of stem cells through upregulation of the various cancer cell surface markers or pluripotent embryonic stem cell markers and activation of various signaling pathways, which are associated with survival and self-renewal of normal stem cells. Moreover, EMT program activation’s acquisition of cancer stem cell (CSC) is attributed to anticancer drug resistance on cancer cells [3, 5–7]. However, the mechanisms that induce the transition of CSC state and anticancer drug or immunotherapy resistance have remained largely unclear.

As has been well described by many studies, neurotrophin receptor TrkC as tyrosine kinase plays an important role in the development of sensory neurons [8]. In addition, a number of studies suggest that TrkC upregulated in various solid tumors or blood cancer and also enabled cancer cells to acquire cellular properties associated with tumorigenicity and metastasis through the regulation of diverse cellular signaling mechanisms [9–13]. However, independent of these observations, in recent years, some of the studies have suggested that TrkC as a dependence receptor might play a functional role as a tumor suppressor in colorectal cancer (CRC) [14] and neuroblastoma [15]. Nonetheless, the role of TrkC as a tumor suppressor in the pathogenesis of CRC has been limited by our finding that TrkC enhances the tumorigenicity and metastasis of CRC by activation of AKT signaling and suppression of transforming growth factor (TGF)-β signaling [16]. In addition, none of the studies has demonstrated whether TrkC serves as a tumor suppressor or oncogene in the pathogenesis of hepatocellular cancer. Furthermore, earlier work did not elucidate the underlying mechanisms by which upregulation of TrkC contributes to the pathogenesis of HCC if TrkC plays a role as a classic oncogene or tumor suppressor.

We present here our findings that delineate how TrkC as oncogene induces the acquisition of CSC traits and chemoresistance via activation of the EMT program by inhibiting degradation of DJ-1, which is one of the key prognostic markers of neurodegenerative diseases such as Parkinson’s disease, Alzheimer’s disease, and Huntington’s disease and various cancer types, including HCC [17, 18].

## Results

### Upregulation of TrkC expression in Hepatocellular carcinoma

**Fig. 1:**
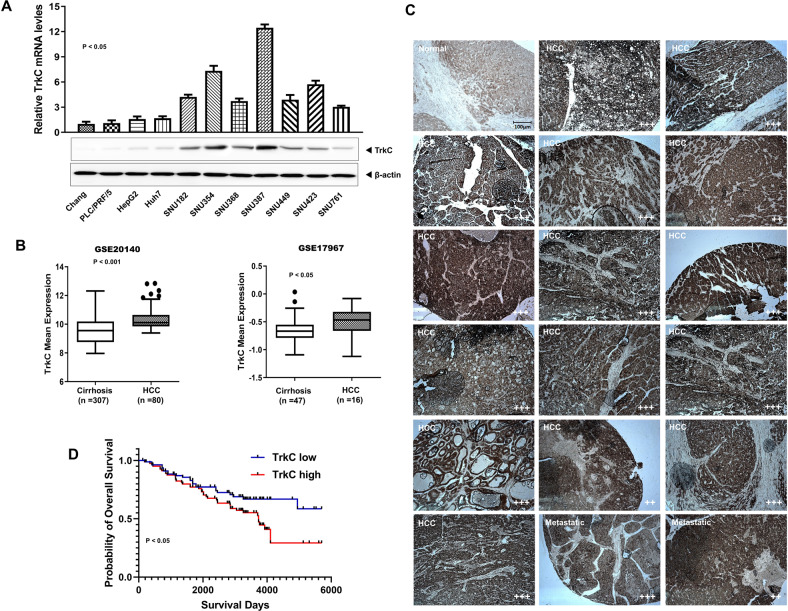
Upregulated TrkC in HCC is correlated with the pathogenesis of HCC. **A** The relative mRNA and protein levels of TrkC in human HCC cancer cells. The mRNA and protein levels of TrkC were normalized to the 18S mRNA and β-actin, respectively. *n* = 3. *P* < 0.05, *t*-test. **B** The mRNA levels of TrkC in cirrhosis and HCC patients from the GSE20140 and GSE17967. *n* = 3. *P* < 0.05, *t*-test. **C** The immunohistochemistry analysis of normal, HCC, and metastatic HCC patients for the TrkC expression (magnification: 200×). The immunohistochemistry intensity of HCC or metastatic HCC was estimated by normal liver tissue, and the staining intensity was scored with grades +~ +++. **D** Kaplan-Meier analysis of overall survival of HCC patients between high or low TrkC expression. The average expression value of TrkC was determined from GSE20140, rank-ordered, and then divided into two equal groups. *P* < 0.05 (*); log-rank test.

Although the effect of TrkC has been identified in other cancers, there is no evidence about the links between TrkC and the pathogenesis of hepatocellular carcinoma (HCC). To evaluate the involvement of TrkC in a functional role of HCC, we began by determining whether HCC cells express the high level of TrkC that could induce pathogenesis of HCC. Real-time RT-PCR (qRT-PCR) and immunoblotting analysis using various HCC cell lines revealed that highly metastatic HCC cells expressed a high level of TrkC relative to Chang cells as a negative control, which lacked the expression of TrkC (Fig. 1A). We next undertook to determine whether the observed upregulation of TrkC in highly metastatic HCC cells was also accompanied by pathogenesis in HCC patients. To do so, we identified the correlation between the TrkC expression and the progression of HCC using the published clinical HCC microarray datasets from Gene Expression Omnibus (GEO) (GSE20140 [19] and GSE17967 [20]). In microarray analysis, we found marked differences in TrkC expression between cirrhosis and HCC patients. TrkC was markedly upregulated in HCC patients relative to patients with cirrhosis (Fig. 1B). To further demonstrate the above observations, we performed immunohistochemistry (IHC) analyses using a series of normal or cancer tissues from 44 HCC patients. IHC analysis revealed that upregulation of TrkC is markedly associated with the pathogenesis of HCC. The tissues of HCC and metastatic HCC patients expressed a high level of TrkC relative to normal samples, which revealed a lack of TrkC expression (Figs. 1C and S1). We next determine whether upregulation of TrkC is directly associated with HCC patient survival. Consistent with the above observations, a high level of TrkC was a powerful predictor of survival outcome. Kaplan-Meier analysis revealed that HCC patients with high levels of TrkC expression resulted in a markedly poorer survival outcome of HCC patients (Fig. 1D), demonstrating that induction of TrkC is critical to the pathogenesis of HCC.

### TrkC is required for the metastatic ability of HCC cells

**Fig. 2:**
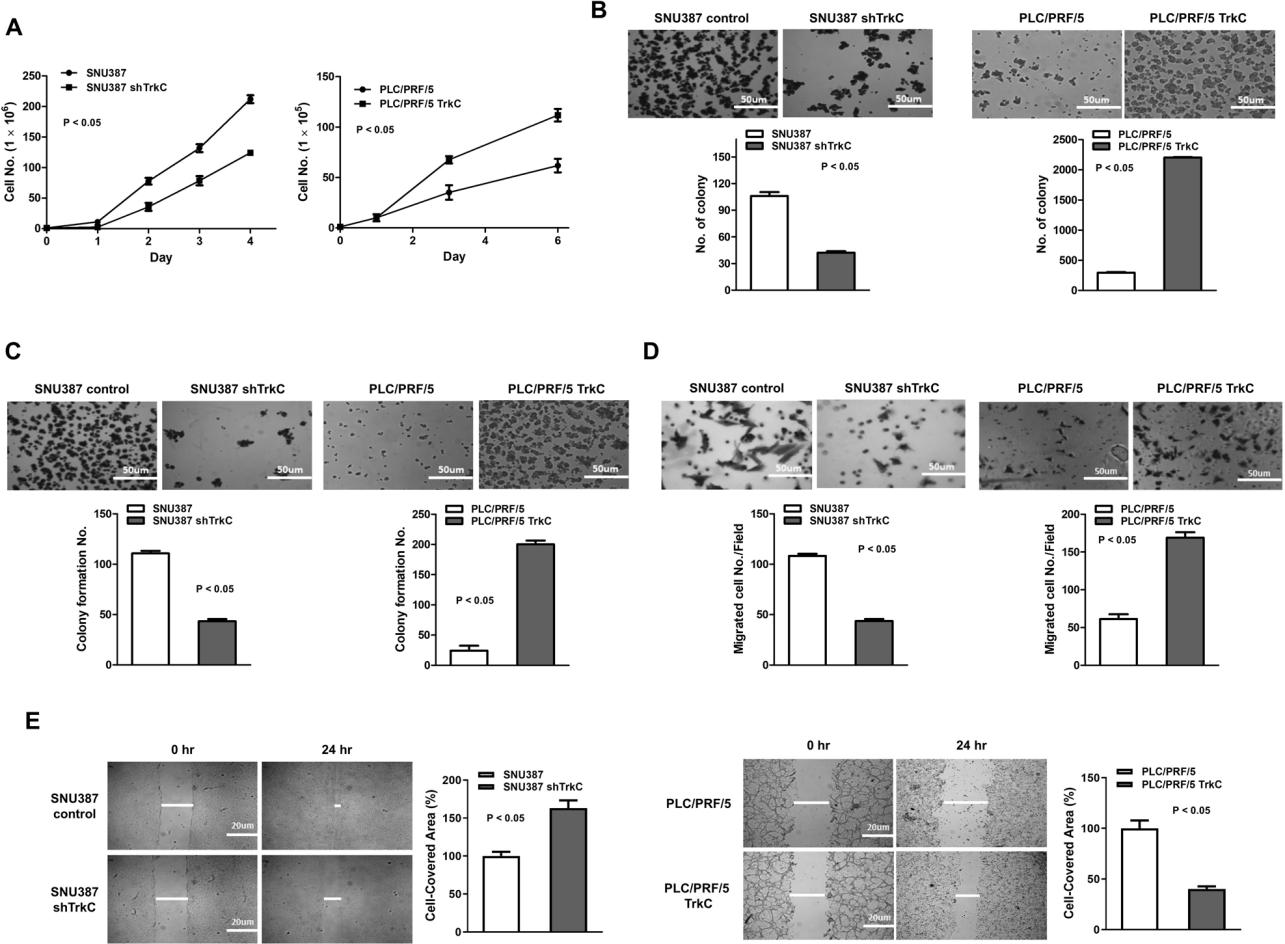
The ability of TrkC to enhance the tumorigenic and metastatic potential of HCC. **A** The cell growth analysis of PCL/PRF/5, PCL/PRF/5 TrkC, SNU387 control-shRNA, or SNU387 TrkC-shRNA cells. *n* = 3. *P* < 0.05, *t*-test. Each bar; mean ± SEM. **B** Phase-contrast images and Soft agar analysis of PCL/PRF/5, PCL/PRF/5 TrkC, SNU387 control-shRNA, or SNU387 TrkC-shRNA cells. *n* = 3. *P* < 0.05, *t*-test. **C** Phase-contrast images and spheroid formation analysis of PCL/PRF/5, PCL/PRF/5 TrkC, SNU387 control-shRNA, or SNU387 TrkC-shRNA cells. *n* = 3. *P* < 0.05, *t*-test. **D** Phase-contrast images and Migration analysis of PCL/PRF/5, PCL/PRF/5 TrkC, SNU387 control-shRNA, or SNU387 TrkC-shRNA cells. *n* = 3. *P* < 0.05, *t*-test. **E** Phase-contrast images and Wound healing analysis of PCL/PRF/5, PCL/PRF/5 TrkC, SNU387 control-shRNA, or SNU387 TrkC-shRNA cells. The wound healing rate was calculated based on the captured images and quantified using the MRI Wound Healing Tool of ImageJ. *n* = 3. *P* < 0.05, *t*-test.

In light of the induction of TrkC in the HCC, we asked whether TrkC might play key functional roles in governing metastatic dissemination of HCC. To address this question, TrkC was knocked down or overexpressed via a pLenti-TrkC plasmid or short hairpin RNA to generate SNU387 HCC cell lines lacking the expression of the TrkC protein and PLC/PRF/5 cell lines overexpressing the corresponding protein (data not shown) and then determine whether the loss or gain of TrkC expression could regulate the growth of HCC cells. SNU387 TrkC knockdown cells significantly reduced cell growth relative to SNU387 control cells. In contrast, this difference was largely reversed by TrkC overexpression in PLC/PRF/5 cells (Fig. 2A). We next determine whether the expression of TrkC could directly regulate the metastatic ability of HCC cells. We observed that colony-forming ability enhanced in the presence of TrkC in PLC/PRF/5 cells. In contrast, the loss of TrkC expression leads to failing to increase the colony formation of SNU387 cells (Fig. 2B). To further confirm the direct effects of TrkC on an acquisition of anchorage independence, we conducted an anoikis analysis. We found that upregulation of TrkC enhanced the acquisition of anchorage independence but decreased expression of TrkC was accompanied by significant decreases in the sphere formation of SNU387 cells (Fig. 2C). As an anoikis analysis, we next tested the effect of TrkC in HCC cell migration and invasion ability. We found that PLC/PRF/5-TrkC and SNU387 control-shRNA cells had significantly increased migration and invasion ability relative to their corresponding cells (Fig. 2D, E), confirming the direct effects of TrkC on cell migration and invasion of HCC cells. Taken together, these observations indicated that upregulation of TrkC is essential for directly maintaining the tumorigenic and metastatic traits of HCC cells to acquire successful colonization of distant tissues.

### TrkC promotes tumorigenicity and metastasis of HCC cells in vivo

**Fig. 3:**
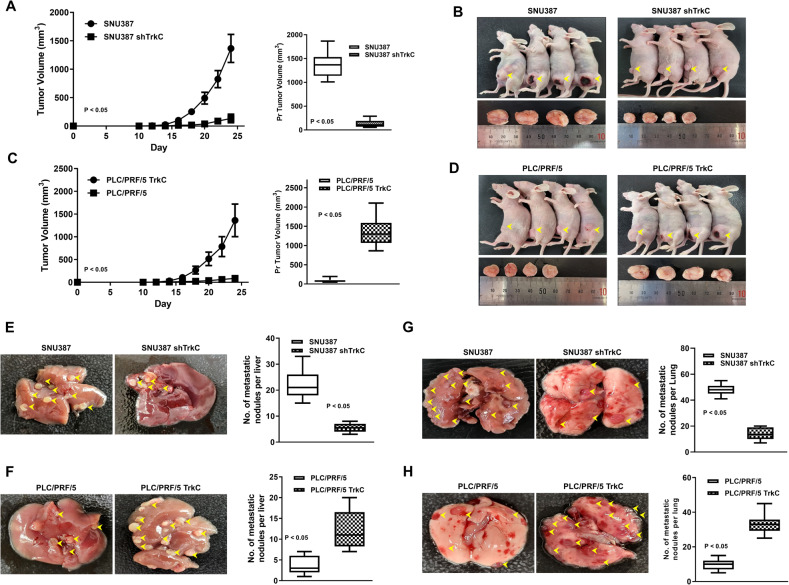
Upregulation of TrkC increased tumor initiation and metastasis of HCC cells in vivo. In vivo tumor formation (**A**) and the representative images (**B**) of control SNU387 or TrkC knockdown cells in the mammary fat pads of mice. Each bar; mean ± SEM.; *P* < 0.05; *t*-test. *n* = 10 mice/group. In vivo tumor formation **C** and the representative images **D** of control PLC/PRF/5 or TrkC overexpression cells in the mammary glands of mice. The error bar, mean ± SEM; *P* < 0.05; *t*-test. *n* = 10 mice/group. **E** Quantification and representative images of liver surface metastatic foci of control SNU387 or TrkC knockdown cells. *P* < 0.05; *t*-test. *n* = 10 mice/group. (**F**) Quantification and representative images of liver surface metastatic foci of control PLC/PRF/5 or TrkC overexpression cells. *P* < 0.05; *t*-test. *n* = 10 mice/group. **G** Quantification and representative images of lung surface metastatic foci of control SNU387 or TrkC knockdown cells. *P* < 0.05; *t*-test. *n* = 10 mice/group. **H** Quantification (upper) and representative images (lower) of lung surface metastatic foci of control PLC/PRF/5 or TrkC overexpression cells. *P* < 0.05; *t*-test. *n* = 10 mice/group.

Induced TrkC expression plays a pivotal role in enhancing metastatic potential in HCC cells, causing us to also focus on maintaining the tumorigenicity and metastatic dissemination in vivo via upregulation of TrkC. To determine whether the loss or gain of TrkC expression could affect primary tumor formation in vivo, we injected SNU387 cells expressing TrkC shRNA or TrkC-expressing PLC/PRF/5 cells into the mouse and observed that tumors arising from SNU387 TrkC-shRNA cells had significantly reduced in size and tumor growth relative to those arising from its control cells (Fig. 3A, B). However, this difference was completely reversed when TrkC-expressing PLC/PRF/5 cells were injected into mice. PLC/PRF/5 TrkC cells were significantly increased growth rate and tumor size as efficiently as SNI387 control-shRNA cells (Fig. 3C, D).

In addition, we found that tumors arising from liver metastases by PLC/PRF/5 TrkC and SNU387 control-shRNA cells had significantly reduced relative to its control cells (Fig. 3E, F). Also, The number of metastatic nodules in the lung in PLC/PRF/5 TrkC and SNU387 control-shRNA cells increased 32-fold and 12-fold than the mice bearing PLC/PRF/5 and SNU387 TrkC-shRNA cells, respectively (Fig. 3G, H). These observations demonstrate that the expression of TrkC is essential for both the tumorigenicity and metastasis of HCC.

### TrkC significantly activates STAT3 via inducing DJ-1 stability

**Fig. 4:**
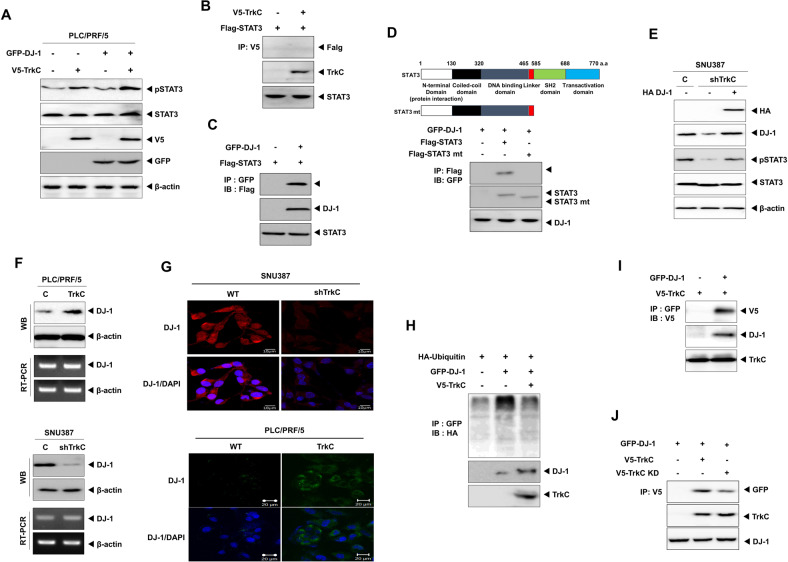
TrkC activates the STAT3 pathway via inhibition of the DJ-1 degradation. **A** Immunoblot analysis of phospho-STAT3, STAT3, TrkC, and DJ-1 in PLC/PRF/5 after transfected with DJ-1 or TrkC. β-actin as a loading control. **B** Identification of complex formation of TrkC/STAT3. **C** Identification of complex formation of DJ-1/STAT3 by immunoprecipitation. **D** Identification of the STAT3 region that interacted with DJ-1. **E** Immunoblot analysis of phospho-STAT3, STAT3, and DJ-1 in SNU387 TrkC-shRNA cells after transfected with DJ-1. β-actin as a loading control. **F** Immunoblot and RT-PCR analysis of DJ-1 in PLC/PRF/5, PLC/PRF/5 TrkC, SNU387 control-shRNA, and SNU387 TrkC-shRNA cells. β-actin as a loading control. **G** Immunofluorescence analysis of DJ-1 in PLC/PRF/5, PLC/PRF/5 TrkC, SNU387 control-shRNA, and SNU387 TrkC-shRNA cells. The nuclei of cells were stained with DAPI (blue). **H** Immunoprecipitation analysis of 293 T cells transfected with the ubiquitin, GFP-DJ-1, and V5-TrkC constructs, as indicated. **I** Identification of complex formation of DJ-1/TrkC by immunoprecipitation. **J** Identification of the effect of the kinase activity of TrkC in the complex formation of TrkC/DJ-1.

DJ-1 has been reported negative regulator of PTEN (Phosphatase And Tensin Homolog) via reducing expression of PTEN [21] and plays a central regulator of astrogliosis in brain injury via activation of STAT3 [22], which also plays a critical role in cancer pathogenesis [23]. In our previous study, TrkC-mediated JAK2 activation promotes STAT3 activation [24]. However, earlier works did not reveal the underlying mechanism of cross-talk between TrkC and DJ-1 contributing to STAT3 activation in cancer progression. To investigate this possibility, we initially interrogate whether TrkC or DJ-1 induces activation of STAT3 by increasing STAT3 expression. We found no significant differences in mRNA or protein levels and luciferase activity of STAT3 by expression of TrkC or DJ-1 (Figs. S2A, B, and 4A). In contrast, ectopic expression of TrkC and DJ-1 increases the phosphorylation of STAT3, respectively, and collaboration of TrkC and DJ-1 more strongly induced its activation (Fig. 4A). We next determine whether the tyrosine kinase activity of TrkC is required for the STAT3 activation to promote tumorigenicity and metastasis of HCC. These data demonstrated that TrkC or DJ-1 might induce STAT3 activation through interaction with STAT3, and the tyrosine kinase activity of TrkC is essential for activating STAT3 in the presence of DJ-1. To identify this possibility, we asked whether TrkC or DJ-1 positively regulates STAT3 activation by interaction with STAT3. Surprisingly, STAT3 directly interacted with DJ-1 but not TrkC (Fig.  4B, C). In addition, the region of STAT3 containing SH2 and the transactivation domain is essential for the formation of the STAT3/DJ-1 complex (Fig. 4D). These observations suggested that TrkC is involved in the activation of STAT3 indirectly.

So, we proceeded to determine the functional role of TrkC in regulating STAT3 activation. We found that the level of STAT3 phosphorylation was markedly reduced in SNU387 TrkC-shRNA cells, but the expression of DJ-1 efficiently recovered STAT3 activation. Most strikingly, we found that decreased TrkC expression in SNU387 cells significantly reduced DJ-1 expression and STAT3 activation (Fig. 4E). Also, we identified these results using the lungs of mouse experiments. The expression of DJ-1 and TrkC or the activation of STAT3 in lungs of PLC/PRF/5 TrkC and SNU387 control-shRNA cells had significantly induced, but STAT3 expression did not change relative to its control cells, respectively (Fig. S3). We then tested whether the reduction of DJ-1 expression is due to direct translational repression by the downregulated TrkC. Interestingly, there are no marked differences in mRNA levels of DJ-1 by TrkC expression. In contrast, by immunoblotting and immunofluorescence analysis, we found that the levels of DJ-1 markedly increased by the upregulation of TrkC (Fig. 4F, G). These observations indicate that TrkC might induce the stability of DJ-1 via inhibiting the ubiquitin-proteasome pathway-mediated DJ-1 degradation to induce STAT3 activation. Indeed, upregulation of TrkC expression leads to activation of the STAT3 signaling pathway via the induction of DJ-1 stability (Fig. 4H). Therefore, it is possible that TrkC may interact with DJ-1 to inhibit the ubiquitination of DJ-1. Consistently, we found that TrkC directly interacted with DJ-1 (Figs. 4I, S4A, B). However, ectopic TrkC kinase-dead expression significantly decreases the phosphorylation of STAT3 via reduction of TrkC/DJ-1 complex formation relative to the introduction of its wild-type (Figs. 4J and S5), suggesting that the tyrosine kinase activity of TrkC is required for the activation of STAT3 by inducing the TrkC/DJ-1 complex formation. Also, these observations provided the first direct indication that the acquisition of malignant traits of HCC cells depends on HCC cells’ ability to express a high level of TrkC and TrkC-mediated inhibition of DJ-1 degradation.

### Increased DJ-1 stability by TrkC is essential for maintaining states of cancer stem cells

**Fig. 5:**
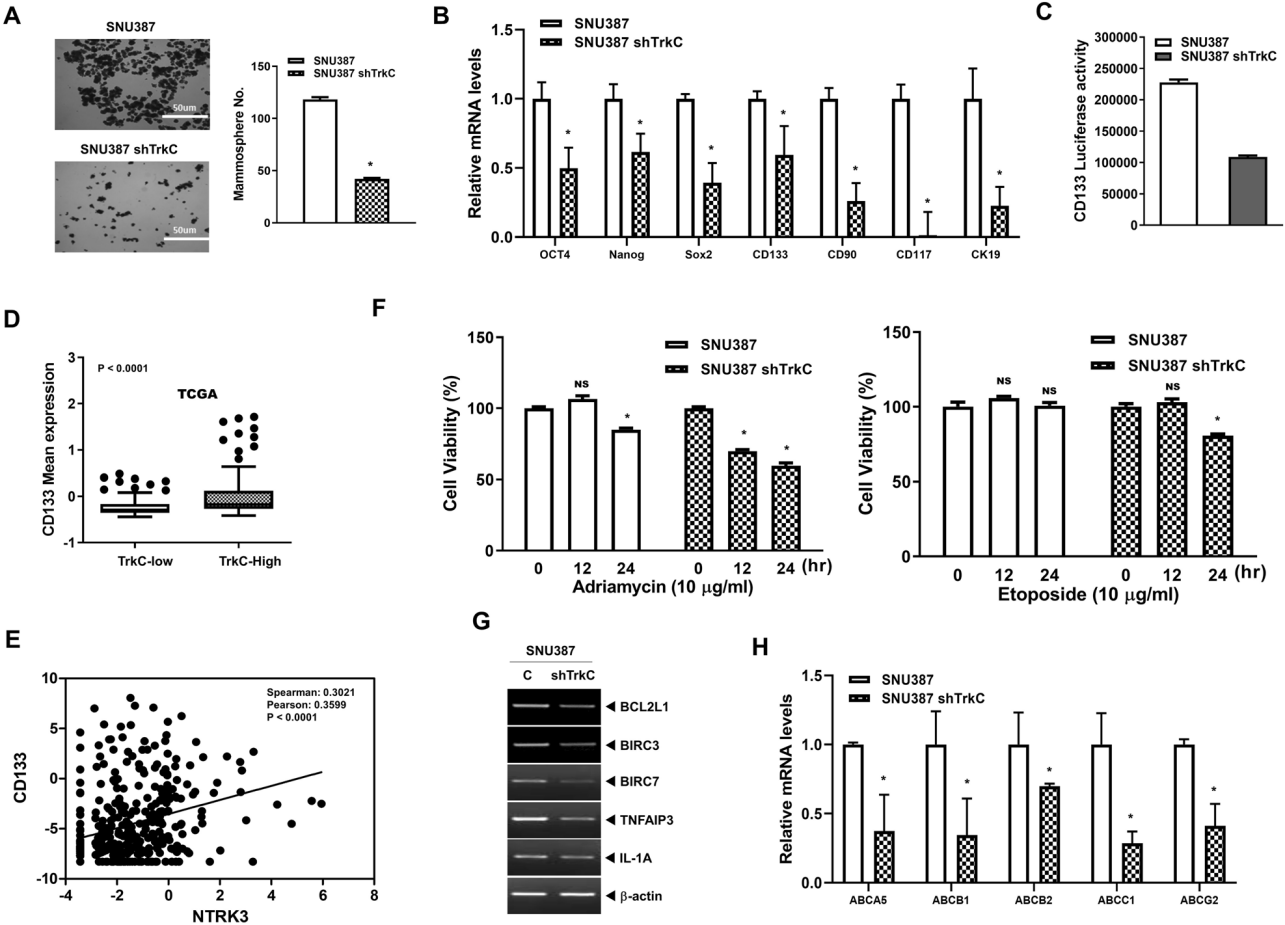
Contribution of TrkC in the acquisition of cancer stem cells of HCC cells. **A** Phase-contrast images and quantification of spheroid formation of SNU387 control- or TrkC-shRNA cells. **B** Quantitative RT-PCR analysis of human embryonic stem (hES) cells markers (Oct4, Nanog, Sox2) and specific CSC markers (CD90, CD117, CD133, and CK19) of HCC in control SNU387 or TrkC knockdown cells. The mRNA level of indicated markers were normalized to The 18 S mRNA. *n* = 3. *P* < 0.05, (*) relative to SNU387; *t*-test. **C** The Luciferase reporter assay for transcriptional activity of CD133 in control SNU387 or TrkC knockdown cells. *n* = 3. Each bar: ± SEM. *P* < 0.05, *t*-test. **D** The relative mRNA expression of CD133 in human HCC patients between high or low TrkC expression. The average expression value of CD133 from the TCGA datasets was determined, rank-ordered, and then divided into two equal groups. P < 0.0001 relative to TrkC-Low; *t*-test. **E** The correlation of TrkC and CD133 expression in TCGA datasets. *P* < 0.0001; spearman’s correlation coefficient. **F** Cell viability analysis in SNU387 control- or TrkC-shRNA cells with treatment of Adriamycin and Etoposide. *n* = 3. P < 0.05 (*) relative to no treatment; NS: not significant; *t*-test. **G** The mRNA levels of an*t*i-apoptotic markers (BCL2L1, BIRC3, BIRC7, TNFAIP3, and IL-1A) in control SNU387 or TrkC knowdown cells. β-actin as a loading control. **H** The relative mRNA expression of ABC transporters (ABCA5, ABCB1, ABCB2, ABCC1, and ABCG2) in control SNU387 or TrkC knockdown cells. *n* = 3. *P* < 0.05 (*) relative to SNU387; *t*-test.

These above observations led us to investigate whether the generation and maintenance of CSCs might depend on the activation of STAT3, which promotes cancer stemness-mediated chemoresistance [23, 25], by TrkC-mediated inhibition of DJ-1 degradation. To address this question, we determine whether the acquisition of spheroid-forming ability in HCC, which is associated with the existence of human CSCs and tumor-initiating cells (TICs) [26, 27], correlated with activation of STAT3 by TrkC-mediated inhibition of DJ-1 degradation. Interestingly, TrkC significantly enhances the spheroid forming ability of cancer cells. The spheroid forming ability of SNU387 and PLC/PRF/5 TrkC cells increased by approximately 2.8-fold and 33-fold relative to its TrkC knockdown and parental cells, respectively (Figs. 5A and S6A). We next examined increased TrkC expression forced to arising and maintaining CSCs via inducing pluripotent embryonic stem cell markers such as Oct4, Nanog, SOX2, and CSC surface markers (CD133, CD90, CD117, and CK19) of HCC, which are essential for self-renewal, metastasis, recurrence, chemoresistance of CSC [6, 28–31]. In the qRT-PCR analysis, SNU387 TrkC-shRNA cells and PLC/PRF/5 parental cells significantly upregulated the expression of mRNAs encoding embryonic stem cell markers and CSC surface markers of HCC relative to SNU387 and PLC/PRF/5 TrkC cells (Figs. 5B and S6B).

Specifically, the activation of Src is required for the upregulation of CD133 to induction of epithelial-mesenchymal transition by increasing N-cadherin expression [32]. Independent of this finding, our previous finding demonstrated that TrkC-mediated c-Src activation leads to activating the JAK2/STAT3 pathway [24]. Therefore, we further assess whether TrkC-mediated STAT3 activation leads to the upregulation of CD133. We found marked upregulation of CD133 luciferase activity in SNU387 and PLC/PRF/5 TrkC cells relative to that of SNU387 TrkC-shRNA and PLC/PRF/5 cells (Figs. 5C and S6C). Once again, we found that the HCC patients with a high level of TrkC revealed upregulation of CD133, CD117, CK19, and CD90 expression (Figs. 5D, S7A, B) from the TCGA dataset and GSE20140 [19] relative to HCC patients with a low TrkC expression. Also, TrkC expression is significantly associated with CD133, CD117, CK19, and CD90 expression from the TCGA dataset and GSE20140 [19] (Figs. 5E, S8A, B). These observations indicated that TrkC-mediated upregulation of cellular markers closely associated with normal and cancer stem cells was accompanied by accumulating the CSC population of HCC.

Cancer cell populations with the properties of CSCs play a pivotal role in resistance to conventional chemotherapy [1, 5, 6], causing us to also focus on the possible effect of TrkC-mediated inhibition of DJ-1 degradation in chemoresistance. We observed that upregulation of TrkC revealed elevated resistance to adriamycin and etoposide treatments at the period of the same time by upregulating transcript levels of anti-apoptotic genes (such as BCL2L1, BIRC3, BIRC7, TNFAIP3, IL-1A) relative to its TrkC knockdown cells (Fig. 5F, G). To provide further evidence that the elevated resistance to chemotherapy drugs was mediated by upregulation of TrkC expression, we investigated the effects of TrkC on chemoresistance by analyzing the expression of ATP-binding cassette (ABC) transporters, which are multidrug resistance proteins highly attributed to the drug resistance of CSCs [33]. The level of ABCA5, ABCB1, ABCB2, ABCC1, and ABCG2 was significantly decreased in SNU387 TrkC-shRNA cells (Fig. 5H). In contrast, these mRNA levels were markedly reversed by TrkC overexpression (Fig. S9). Also, there was no change in mRNAs encoding ABCA1 and ABCA2 by loss or gain of TrkC (Data not shown). Moreover, these mRNA levels by both introductions of TrkC and DJ-1were markedly increased relative to the expression of TrkC or DJ-1 (Fig. S10G).

Previous studies have reported the correlation between DJ-1 and the transition of stem cells or tumor-initiating cancer stem cells (CSC), and DJ-1 induces and maintains CSC states by protecting EGFR degradation [34, 35]. Also, DJ-1 positively regulates liver progenitor cell (LPC) expansion by enhancing the formation of LPC-associated fibrosis and inflammatory niches via induction of pro-inflammatory cytokines (IL-6 and TNF-α) and recruiting macrophages [36]. These observations suggested that TrkC-mediated induction of DJ-1 stability works to induce CSC state through STAT3 activation. So, we further examine whether DJ-1 played a critical role in the TrkC-mediated induction of SCS state. The downregulation of mRNA levels or luciferase activity of embryonic stem (Oct4, Nanog, Sox2), CSC surface markers, and ATP-binding cassette (ABC) transporters was prevented by introducing DJ-1 into SNU387 TrkC-shRNA cells (Fig. S10A–C). Also, both DJ-1/TrkC expression significantly increases the mRNA level and luciferase activity of embryonic stem cell markers, CSC surface markers, and ATP-binding cassette (ABC) transporters than the introduction of TrkC or DJ-1 (Fig. S10D–G). However, the knockdown of DJ-1 in PLC/PRF/5 DJ-TrkC cells markedly reduces the mRNA level of embryonic stem cell markers, CSC surface markers, and ATP-binding cassette (ABC) transporters relative to both introduction of TrkC and DJ-1 (Fig. S11A–C).

### Prevention of DJ-1 degradation by TrkC leads to induction of the EMT program

**Fig. 6:**
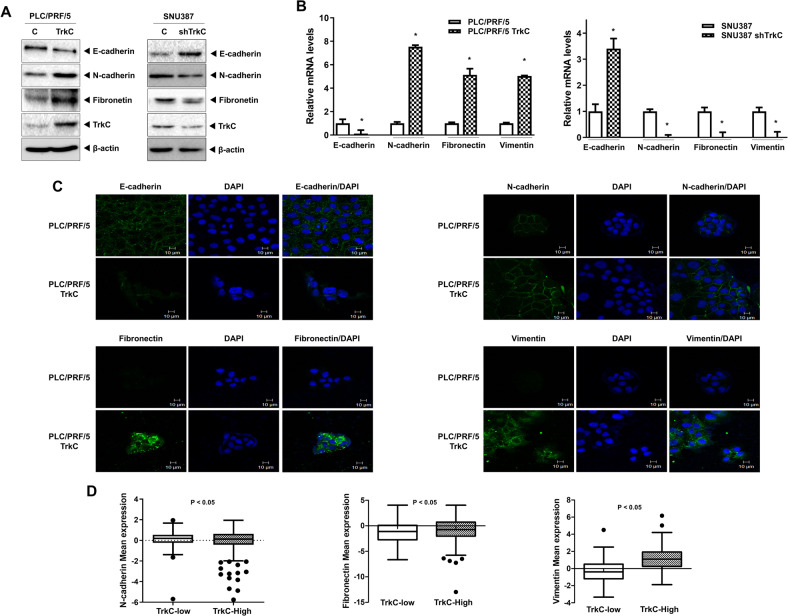
Effect of TrkC in the activation of the EMT program in HCC cells. **A** Immunoblot analysis of E- and N-cadherin, and fibronectin, in PLC/PRF/5, PLC/PRF/5 TrkC, SNU387 control-shRNA, and SNU387 TrkC-shRNA cells. β-actin as a loading control. **B** The relative mRNA levels of E- and N-cadherin, fibronectin, and vimentin in PLC/PRF/5, PLC/PRF/5 TrkC, SNU387 control-shRNA, and SNU387 TrkC-shRNA cells. *n* = 3. *P* < 0.05 (*) relative to SNU387; *t*-test. **C** Immunofluorescence analysis of indicated mesenchymal (fibronectin, vimentin, and N-cadherin) and epithelial (E-cadherin) markers in PLC/PRF/5 or TrkC overexpression cells. The nuclei of cells were stained with DAPI (blue). **D** The relative mRNA expression of indicated mesenchymal markers (N-cadherin, fibronectin, and vimentin) in HCC patients between high or low TrkC expression. The average expression value of indicated EMT-related markers from the TCGA datasets was determined, rank-ordered, and then divided into two equal groups. *P* < 0.0001 relative to TrkC-Low; *t*-test.

The acquisition of CSC-mediated chemoresistance has been associated with the EMT program in the recurrence and metastasis of tumors [2, 6]. Therefore, we determine whether the observed TrkC-mediated upregulation of DJ-1 stability to enhance chemotherapy resistance was also accompanied by the induction of the EMT program. To test this possibility, we initially examined whether TrkC expression in HCC cells forces to undergo the EMT program. Ectopic expression of TrkC in PLC5/PRF/5 cells, which is weakly tumorigenic HCC cells, significantly increases the expression of mesenchymal markers (N-cadherin, fibronectin) but markedly reduces the expression of an epithelial marker such as E-cadherin. In contrast, the loss of TrkC from SNU387 cells resulted in reversing the expression pattern of N-cadherin, fibronectin, E-cadherin proteins relative to control cells (Fig. 6A). Also, we confirmed our observation by quantitative RT-PCR and immunostaining analysis. We found that upregulation of TrkC led to induction of N-cadherin, fibronectin, vimentin, and reduction of E-cadherin mRNA and protein expression. However, a reversed expression pattern of mRNA and proteins encoding epithelial and mesenchymal markers was observed in SNU387 control-shRNA cells relative to control cells (Figs. 6B, C, and S12). To provide further evidence that TrkC induces the EMT program, we determined whether upregulation of TrkC is directly associated with the induction of expression of mesenchymal markers. Consistent with our above observations, HCC patients with high TrkC expression elicited the expression of mesenchymal markers such as N-cadherin, fibronectin, and vimentin markedly induced during the EMT program (Fig. 6D), demonstrating that TrkC expression is essential for induction of the EMT program. Also, both DJ-1/TrkC expression significantly increases the mRNA level of mesenchymal markers and decreases E-cadherin expression relative to the introduction of TrkC or DJ-1 (Fig. S13A, B). Moreover, this induction of the mRNA level of mesenchymal markers and reduction of E-cadherin expression in PLC/PRF/5 TrkC-DJ-1 cells was reversed by introducing DJ-1 shRNA cells (Fig. S13C).

**Fig. 7:**
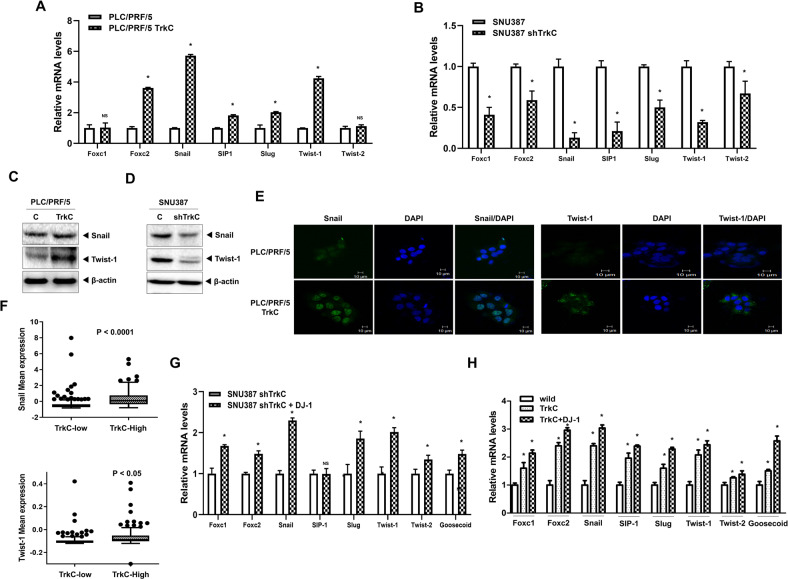
Upregulated TrkC induces the EMT program via induction of EMT-TFs. **A** The relative mRNA levels of EMT-TFs (Foxc1, Foxc2, Snail, SIP1, Slug, Twist-1, Twist-2, and Goosecoid) in PLC/PRF/5 or PLC/PRF/5 TrkC cells. *n* = 3. *P* < 0.05 (*) relative to PLC/PRF/5; NS: not significant; *t*-test. **B** The relative mRNA levels of EMT-TFs in control SNU387 or TrkC knockdown cells. *n* = 3. *P* < 0.05 (*); NS: not significant; *t*-test. **C** Immunoblo*t* analysis of Snail and Twist-1 in PLC/PRF/5 or PLC/PRF/5 TrkC cells. β-actin as a loading control. **D** Immunoblot analysis of Snail and Twist-1 in control SNU387 or TrkC knockdown cells. β-actin as a loading control. **E** Immunofluorescence analysis of Snail and Twist-1 in PLC/PRF/5 or PLC/PRF/5 TrkC cells. The nuclei of cells were stained with DAPI (blue). **F** The relative mRNA expression of Twist-1 and Snail in human HCC patients between high or low TrkC expression. The average expression value of Twist-1 and Snail from the TCGA datasets was determined, rank-ordered, and then divided into two equal groups*. P* < 0.0001, *t*-test. **G** The relative mRNA levels of EMT-TFs in SNU387 TrkC-shRNA cells transfected with or without DJ-1. *n* = 3. *P* < 0.05 (*); NS: not significant; *t*-test. **H** The relative mRNA levels of EMT-TFs in PLC/PRF/5 cells transfected with or without DJ-1 or TrkC. *n* = 3. *P* < 0.05 (*); NS: not significant; *t*-test.

To understand more precisely how upregulation of TrkC led to the induction of the EMT program, we further assessed the functional role of TrkC in the activation of EMT-inducing transcription factors (EMT-TF). Ectopic expression of TrkC in PLC5/PRF/5 cells significantly increases the expression of mRNAs encoding EMT-TFs such as Foxc2, Snail, SIP1, Slug, Twist-1 relative to control cells, whereas the levels of Foxc1 and Twist-2 did not change in expression. In contrast, the loss of TrkC from SNU387 cells resulted in reversing the expression pattern of EMT-TFs, including Foxc1 and Twist-2, relative to control cells (Fig. 7A, B). Next, we confirmed the above observation by immunoblot and immunostaining analysis. Furthermore, induced TrkC expression significantly upregulated various EMT-TFs such as snail and Twist-1 (Figs. 7C–E, and S14). To support our observations, we assessed the direct association between the expression of these EMT-TFs and induced TrkC expression in HCC patients using the TCGA dataset. Consistent with our above results, the level of mRNAs encoding EMT-TFs was markedly elevated in HCC patients with high TrkC expression relative to HCC patients with low level of TrkC (Figs. 7F and S15). We also found that induction of the EMT program is a direct consequence of TrkC-mediated activation of EMT-TFs by correlation analysis using the TCGA dataset (Fig. S16). We next determine whether TrkC-mediated induction of DJ-1 stability leads to the upregulation of EMT-TFs. The introduction of DJ-1 into SNU387 TrkC-shRNA cells can activate the expression of a large number of EMT-TFs, except SIIP-1 (Fig. 7G). Moreover, both TrkC and DJ-1 markedly induce the expression of EMT-TFs in PLC/PRF/5 (Fig. 7H). In addition, this induction of the expression of EMT-TFs in PLC/PRF/5 TrkC-DJ-1 cells was markedly reduced by introducing DJ-1 shRNA cells (Fig. S17), indicating that induction of the EMT program by EMT-TF was mediated directly by activating STAT3 via TrkC or TrkC-mediated inhibition of DJ-1 degradation.

## Discussion

Our findings suggest that the acquisition of aggressive traits of HCC, including metastatic dissemination, is strongly dependent on the upregulation of TrkC. We present here that TrkC is significantly upregulated in HCC cells and tumor or metastatic HCC patients. Also, the malignant progression of HCC in vivo and in vitro is closely associated with the upregulation of TrkC.

Previous work suggested that upregulation of DJ-1 regulates astrogliosis by STAT3 activation [22] and leads to acquiring chemoresistance of cancer cells [37, 38]. However, it remains unclear the signaling mechanisms that maintain the stability of DJ-1 by TrkC and activate STAT3 by DJ-1 in cancer. In our hands, upregulation of TrkC significantly induces the stabilization of DJ-1 via direct interaction and then leads to more strong activation of STAT3 through the induction of DJ-1-STAT3 complex formation. Furthermore, TrkC itself can induce the phosphorylation of STAT3 by JAK2-TrkC interaction [24], suggesting that TrkC activates STAT3 through diverse signaling pathways.

Together, STAT3 activation by TrkC or TrkC-mediated DJ-1 suggested a possible link between the acquisition of self-renewal capacity and resistance to chemotherapeutic drugs. In our work, the subpopulation of mesenchymal/stem cells increased the upregulation of stem cell markers (Sox2, Nanog, and Oct4) and specific CSC markers (CD133, CD90, CD117, and CK19) of HCCs by TrkC-mediated inhibition DJ-1 degradation. Also, upregulation of DJ-1 expression by TrkC renders HCC cells more resistant to adriamycin (doxorubicin) and etoposide treatments by inducing the expression of multiple members of the ABC transporters. These inductions of ABC transporters promote tumor progression, recurrence, induction of self-renewal properties, and overcoming therapeutic agents resistance [4–6].

Although our observations revealed that elevated TrkC expression in HCC had been associated with increased malignant progression, resistance to therapeutic drugs, and more CSC subpopulations in HCC, the molecular mechanism of acquiring drug resistance by TrkC is also still required. Responding to this question, our discovery presented here that induction of TrkC significantly increases drug resistance via generating a subpopulation of cells with the self-renewing cells by activating STAT3 via inhibition of DJ-1 degradation, and the biological concepts, which comes from the previous studies provide direct support our findings. STAT3 activation leads to upregulation of CD90 [39], CD133 [40], CD117 [41], and CK19 [42] to promote stemness and chemoresistance.

Acquisition of CSC-mediated clinical relapse by activation of the EMT program has significantly resistant to conventional chemotherapy and radiotherapy [43, 44]. Also, the malignant progression of cancer is closely associated with the induction of the EMT program, which is a key developmental program and induction of EMT program driven by interactions of EMT-associated autocrine or paracrine signaling, transcriptional factors, and downstream effectors [1, 2, 45]. In this work, we showed that induced TrkC is necessary to induce the EMT program in HCC to govern metastatic dissemination. This finding suggests that the upregulation of TrkC can directly regulate the activation of the EMT program.

After cancer cells have passed through the EMT program, these cells enhance the malignancies by inducing and maintaining their populations of CSC through induction of EMT-TFs, such as Snail, Slug, SIP-1, Gosecoid, ZEB1, Foxc1, Foxc2, Twist-1, and Twist-2 involved in activation of EMT program through the autocrine signaling and other diverse stimuli [4, 46]. Responding to these previous results, we suggest that TrkC ensures the induction of the EMT program for the acquisition of mesenchymal and basal traits. Thus, we observed that induced TrkC increased the expression of master EMT-TFs, notably Foxc1, Foxc2, Snail, SIP1, Slug, Twist-1, and Twist-2. In addition to the acquisition of self-renewal ability, we observed that activation of EMT by TrkC-mediated upregulation of EMT-TFs is sufficient for essential for protecting HCC cells from therapeutic agents.

Thus, our findings describe the unexpected coverage of how TrkC-mediated inhibition of DJ-1 degradation activates STAT3 to acquire CSC traits and chemoresistance. Taken together, the present work indicates that the upregulation of TrkC in the progression of HCC contributes to the entering CSC state and chemoresistance through activation of EMT. As a result, functional loss of TrkC through the clinical utility of inhibiting tyrosine kinase activity may lead to significantly improved treatment for chemoresistance and radioresistance.

## Experimental Procedures

### Cell culture

All cell lines were maintained as previously [47, 48]. The human HCC cell lines (PLC/PRF/5, Chang, HepG2, HUH7, SNU182, SNU354, SNU368, SNU387, SNU449, SNU423, and SNU761) were cultured in DMEM medium (Gibco, Grand Island, NY) containing 10% adult bovine serum with 1× penicillin-streptomycin. For anticancer drug treatment, cell lines were treated with 10 μg/ml adriamycin and etoposide for a total period of time (Sigma-Aldrich).

### Mice

BALB/c Nu/Nu mice were obtained from Dae Han Bio-Link. All animals were five weeks old female mice and maintained in compliance with the guidelines and protocols approved by the Institutional Animal Care and Use Committee (IACUC) of Gachon University (Approval No. LCDI-2022-0074).

### In Silico Analysis of Clinical Microarray Data

The gene signatures (TrkC, Twist-1, Snail, CD133, CD117, CD90, and CK19) in the published clinical microarray such as GSE14323 [49], GSE17967 [20], GSE5975 [50], and TCGA from HCC patients were extracted and averaged, then In silico analysis was performed as previously described [51]. The boxplot graphs were plotted with gene expression using GraphPad Prism v 5.0 (GraphPad Software, Inc.), and *P* < 0.05 was considered statistically significant.

### Knockdown of DJ-1

pLKO.1 lentiviral plasmids encoding shRNAs targeting the DJ-1 gene were obtained from Sigma-Aldrich (TRCN0000004920) (Table S1). The knockdown of DJ-1 in PLC/PRF/5 TrkC-DJ-1 cells was obtained after infection with lentivirus as previously described [35].

### Immunoblotting, immunohistochemistry, immunofluorescence analysis

All analyses were performed as described previously [10, 52]. For immunoblotting analysis, whole-cell lysates were made using a RIPA lysis buffer, resolved on Bis-Tris 10% gel, and transferred to PVDF membranes. The blots were then probed with various primary antibodies. E-cadherin, fibronectin, N-cadherin, alpha-catenin, and vimentin (BD Biosciences); Twist-1 and DJ-1 (Abcam); STAT3 and phospho-STAT3 (Cell Signaling Technology); HA and GFP (Santa Cruz Biotechnology); V5 (Life Technologies); β-actin (Sigma-Aldrich).

For Immunofluorescence staining, 1 × 10^4^ cells were incubated with specific primary antibodies, washed, and then incubated with secondary antibodies (Invitrogen). Images were acquired using a confocal microscope LSM 700 (ZEISS).

For immunohistochemistry, a tissue microarray slide (CSA) was purchased from Super Bio Chips, and the expression of TrkC at the tissue microarray slide was evaluated by using an indicated antibody. In addition, the evaluation of immunostaining intensity was performed and obtained the score by ImageJ software.

### Anchorage-independent cell growth, Matrigel invasion, Soft agar, and wound healing assays

Assays were performed as previously described with modifications [4, 51]. For cell growth, anchorage-independent cell growth, wound healing, and soft agar assays, 1 × 10^5^ cells/well were seeded into six-well culture plates. For the cell migration and invasion assay, 1 × 10^4^ cells/well were seeded into Matrigel invasion chambers, 8 μm (Corning), containing DMEM with 2.5% FBS. For the spheroid assay, 1 × 10^4^ cells/well were seeded into the Ultra-Low attachment multiple-well plate (Corning) in DMEM/F12 medium supplemented with 20 ng/ml FGF (Sigma-Aldrich), 20 ng/ml EGF (Sigma-Aldrich), B27 (GIBCO). After indicated times, cells counted three fields and were visualized via fixed and stained using crystal violet solution (Sigma-Aldrich). The Cell-Covered Area (%) in wound healing analysis was quantified by ImageJ software.

### A luciferase reporter assay

Reporter activities were determined as previously described [51]. 1 × 10^5^ cells per well in 12-well dishes were seeded and cotransfected with 0.5 μg of STAT3 or CD133 reporter gene constructs and 0.5ug of pCMV-β-gal using Lipofectamine 2000 (Invitrogen). The cell lysates were prepared 48 hr after transfection. The luciferase activities were measured with the Enhanced Luciferase Assay Kit (BD Biosciences).

### RNA preparation and Reverse transcriptase PCR (RT-PCR), and Real-time RT-PCR Analysis

RNA preparation, analysis of RT-PCR, and real-time RT-PCR were performed as previously described [51]. The PCR reactions using SYBR Premix Ex Taq Kit (Takara Bio) were data collection was performed using CFX Opus 384 Real-Time PCR system. The primer sequences are listed in the supplemental experimental procedures (Table S1).

### Animal studies

For evaluating tumorigenic ability by TrkC, 1 × 10^6^ cells were injected subcutaneously into the hind flank regions and tail veins of BALB/c nude mice (*n* = 10). Thirty-two days after tumor cell injections, the mice were euthanized and necropsied. For evaluating metastatic ability by TrkC, 1 × 10^6^ cells were injected into the tail vein of BALB/c nude mice (*n* = 10). After 32 days, the mammary tumor and metastasized lung or liver tissues were isolated and collected at the end of the experiment, washed in PBS, and then counted and photographed visible lung and liver nodules. These experiments were approved by the Institutional Animal Care and Use Committee (IACUC) of Gachon University (Approval No. LCDI-2022-0074).

### Statistical Analysis

All data are presented as the means ± SEM. Student’s t-test (two-tailed) and ANOVA were used to compare the groups of the data in the figures for statistical analysis. *P* < 0.05 or *P* < 0.001 were considered statistically significant.

## Supplementary information

Reproducibility-checklist

Supplementary Table 1

Supplementary Figure Legends

Supplementary Figure 1

Supplementary Figure 2

Supplementary Figure 3

Supplementary Figure 4

Supplementary Figure 5

Supplementary Figure 6

Supplementary Figure 7

Supplementary Figure 8

Supplementary Figure 9

Supplementary Figure 10

Supplementary Figure 11

Supplementary Figure 12

Supplementary Figure 13

Supplementary Figure 14

Supplementary Figure 15

Supplementary Figure 16

Supplementary Figure 17

Wester blotting #1

Wester blotting #2

Wester blotting #3

Wester blotting #4

## Data Availability

All data generated and analyzed during this study are included in this published article and its supplementary information file.
