# Correction to: TrkC-mediated inhibition of DJ-1 degradation is essential for direct regulation of pathogenesis of hepatocellular carcinoma

**DOI:** 10.1038/s41419-022-05382-8

**Published:** 2022-11-22

**Authors:** Min Soo Kim, Won Sung Lee, Yeonmi Park, Wook Jin

**Affiliations:** 1 Laboratory of Molecular Disease and Cell Regulation, Department of Biochemistry, School of Medicine, Gachon University, Incheon, 21999 Republic of Korea; 2 Department of Health Sciences and Technology, GAIHST, Gachon University, Incheon, 21999 Korea

**Keywords:** Metastasis, Cell invasion

Correction to: *Cell Death and Disease* 10.1038/s41419-022-05298-3, published online 6 October 2022

The original version of this article contained an error in the affiliations. Won Sung Lee and Yeonmi Park are graduate students preparing for graduation and need to add a new author affiliation (Department of Health Sciences and Technology, GAIHST, Gachon University, Incheon 21999, Korea) in the published paper as a graduation requirement to graduate in February 2023. Both authors are still working at the same institutions indicated in the manuscript’s author affiliation. The authors apologize for the error. The original article has been corrected.

