# Supplementary material for: TrkC-mediated inhibition of DJ-1 degradation is essential for direct regulation of pathogenesis of hepatocellular carcinoma
Source: Cell Death Dis. 2022 Oct 6;13(10):850. doi: 10.1038/s41419-022-05298-3 (PMC9537181; doi:10.1038/s41419-022-05298-3)
Supplement: Supplementary file 2 — Supplementary Table 1 [file 41419_2022_5298_MOESM2_ESM.docx]

**Table S1. Primer sequences for Knockdown, PCR and real-time RT-PCR**

| **Real-time RT-PCR or RT-PCR Primers** | |
| --- | --- |
| **Gene** | **Primers** |
| **Human CD133** | F: 5’- CCTGGGGCTGCTGTTTATTA -3’  R: 5’- TCACCAACAGGGAGATTGCAAA -3’ |
| **Human CD117** | F: 5′- AGAGACTTGGCAGCCAGAAA -3′  R: 5′- TGCCATCCACTTCACAGGTA -3′ |
| **Human CD90** | F: 5’- TCCAGGCCACGGATTTCAT -3’  R: 5’- CCCACTTCTCCTCAAGGTTTGA -3’ |
| **Human CK19** | F: 5’- TGAGCAGGTCCGAGGTTACT -3’  R: 5’- TCTTCCAAGGCAGCTTTCAT -3’ |
| **Human Oct4** | F: 5’- ACATCAAAGCTCTGCAGAAAGAACT -3’  R: 5’- CTGAATACCTTCCCAAATAGAACCC -3’ |
| **Human Nanog** | F: 5’- CAGCTGTGTGTACTCAATGATAGATTT -3’  R: 5’- ACACCATTGCTATTCTTCGGCCAGTTG -3’ |
| **Human SOX2** | F: 5’- AAATGGGAGGGGTGCAAAAGAGGAG -3’  R: 5’- CAGCTGTCATTTGCTGTGGGTGATG -3’ |
| **Human ABCA1** | F: 5’- AACAGTTTGTGGCCCTTTTG -3’  R: 5’- AGTTCCAGGCTGGGGTACTT -3’ |
| **Human ABCA2** | F: 5’- AGCTGCTGCTCTGGAAGAAC -3’  R: 5’- GCCGCTGTGTAGAAGGAGAC -3’ |
| **Human ABCA5** | F: 5’- CCACTGGAGGAAGAAATGGA -3’  R: 5’- AAAGGCTCATGGTGCTCACT -3’ |
| **Human ABCB1** | F: 5’- CTATGCTGGATGTTTCCGGT -3’  R: 5’- GCTTTGGCATAGTCAGGAGC -3’ |
| **Human ABCB2** | F: 5’- ACGTCCACCCTGAGTGATTC -3’  R: 5’- GACACTGATCCCAGAGCAT -3’ |
| **Human ABCC1** | F: 5’- AAGAAAACAGGGAAGCAGCA -3’  R: 5’- GCTCTCTGGGTTTGAAGTCG -3’ |
| **Human ABCG2** | F: 5’- ATCTTGGCTGTCATGGCTTC -3’  R: 5’- TCTTCGCCAGTACATGTTGC -3’ |
| **Human FOXC1** | F: 5’- ACGGCATCTACCAGTTCATC -3’  R: 5’- TCCTTCTCCTCCTTGTCCTT -3’ |
| **Human FOXC2** | F: 5’- GCCTAAGGACCTGGTGAAGC -3’  R: 5’- TTGACGAAGCACTCGTTGAG -3’ |
| **Human E-cadherin** | F: 5’- TGCCCAGAAAATGAAAAAGG -3’  R: 5’- GTGTATGTGGCAATGCGTTC -3’ |
| **Human N-cadherin** | F: 5’- ACAGTGGCCACCTACAAAGG -3’  R: 5’- CCGAGATGGGGTTGATAATG -3’ |
| **Human Fibronectin** | F: 5’- CAGTGGGAGACCTCGAGAAG -3’  R: 5’- TCCCTCGGAACATCAGAAAC -3’ |
| **Human Vimentin** | F: 5’- GAGAACTTTGCCGTTGAAGC -3’  R: 5’- GCTTCCTGTAGGTGGCAATC -3’ |
| **Human Snail** | F: 5’- CCTCCCTGTCAGATGAGGAC -3’  R: 5’- CCAGGCTGAGGTATTCCTTG -3’ |
| **Human SIP1** | F: 5’- TTCCTGGGCTACGACCATAC -3’  R: 5’- TGTGCTCCATCAAGCAATTC -3’ |
| **Human Slug** | F: 5’- GGGGAGAAGCCTTTTTCTTG -3’  R: 5’- TCCTCATGTTTGTGCAGGAG -3’ |
| **Human Goosecoid** | F: 5’- GAAGGTAAAAGCGATTTGGA -3’  R: 5’- ACATCGCCATCACTTTATTG -3’ |
| **Human TrkC** | F: 5’- AACATTTCCGTCACCTTGACTTGT -3’  R: 5’- AATGTCACAGGAGCATGTAAATGG -3’ |
| **DJ-1 shRNA Primers** | |
| **Gene** | **Primers** |
| Human DJ-1 | F: 5’-GTAGCCGTGATGTGGTCATTT-3’  R: 5’-AAATGACCACATCACGGCTAC-3’ |
