# Supplementary material for: TrkC-mediated inhibition of DJ-1 degradation is essential for direct regulation of pathogenesis of hepatocellular carcinoma
Source: Cell Death Dis. 2022 Oct 6;13(10):850. doi: 10.1038/s41419-022-05298-3 (PMC9537181; doi:10.1038/s41419-022-05298-3)
Supplement: Supplementary file 3 — Supplementary Figure Legends [file 41419_2022_5298_MOESM3_ESM.docx]

**Supplementary Figure Legends**

**Figure S1. The comparison of TrkC expression levels in HCC, metastatic tumors, and adjacent normal samples in the same patients.** The immunohistochemistry intensity of normal vs. HCC and HCC vs. metastatic HCC in the same patients was estimated by matched normal, or HCC samples in the same patients, and the staining intensity by ImageJ program was scored with grades + ~ +++.

**Figure S2. Effects of Trk or DJ-1 in STAT3 expression.** The relative mRNA levels (A) and luciferase reporter (B) analysis of STAT3 expression in PLC/PRF/5 cells transfected with or without DJ-1 and TrkC. The mRNA levels of STAT3 were normalized to The 18S mRNA. *P* < 0.05, *t*-test.

**Figure S3. Effects of TrkC in STAT3 activation and DJ-1 expression.** Representative immunohistochemistry images of TrkC, STAT3, DJ-1, and phospho-STAT3 in the lung of PLC/PRF/5, PLC/PRF/5 TrkC, SNU387 control-shRNA, and SNU387 TrkC-shRNA cells. The immunohistochemistry intensity of SNU387 control vs. TrkC shRNA and PLC/PRF/5 vs. PLC/PRF/5 TrkC was estimated by the ImageJ program, and the staining intensity was scored with grades + ~ +++.

**Figure S4.** **TrkC interacts with DJ-1 directly.** (A) Identification of endogenous complex formation of DJ-1/TrkC in PLC/PRF/5 and SNU387 cells by immunoprecipitation. (B) Identification of endogenous complex formation of DJ-1/TrkC in SNU387 and SNU387 TrkC-shRNA cells by immunoprecipitation.

**Figure S5. Contribution of the kinase activity of TrkC in the activation of STAT3 by induction of TrkC/DJ-1 interaction.** Immunoblot analysis of phospho-STAT3, STAT3, and TrkC expression in SNU387 TrkC-shRNA cells after transfected with TrkC and TrkC kinase-dead constructs. β-actin as a loading control.

**Figure S6. Effect of TrkC in the transition of CSCs.** (A) Phase-contrast images and quantification of spheroid formation of PLC/PRF/5 and PLC/PRF/5 TrkC cells. (B) The relative mRNA levels of human embryonic stem (hES) cells markers (Oct4, Nanog, Sox2) and specific CSC markers (CD90, CD117, CD133, and CK19) of HCC in PLC/PRF/5 and PLC/PRF/5 TrkC cells. P < 0.05 (*) relative to PLC/PRF/5; *t*-test. (C) The Luciferase reporter assay for transcriptional activity of CD133 in PLC/PRF/5 and PLC/PRF/5 TrkC cells. Error bar; mean ± SEM. *P* < 0.05, *t*-test.

**Figure S7. Effect of TrkC in the acquisition of cancer stem cells of HCC patients.** The relative mRNA levels of CD133, CD117, CK19, and CD90 expression in human HCC patients between high or low TrkC expression. The average expression value of TrkC from the GSE20140 (A) and TCGA (B) datasets were determined, rank-ordered, and then divided into two equal groups. P < 0.05, 0.005, or 0.0001 relative to TrkC-Low; *t*-test.

**Figure S8. Contribution of TrkC in the transition of cancer stem cells of HCC patients.**  The correlation of TrkC and CD133, CD117, CK19, and CD90 expression in GSE20140 (A) and TCGA (B) datasets. P < 0.0001; spearman's correlation coefficient.

**Figure S9. Trk induces the expression of ABC transporters.** The relative mRNA levels of human ABC transporters (ABCA5, ABCB1, ABCB2, ABCC1, and ABCG2) in PLC/PRF/5 and PLC/PRF/5 TrkC cells. P < 0.05 (*) relative to SNU387; *t*-test.

**Figure S10. Effect of TrkC in the transition of CSCs.** (A) The relative mRNA levels of human embryonic stem (hES) cells markers (Oct4, Nanog, Sox2) and specific CSC markers (CD90, CD117, CD133, and CK19) of HCC in control SNU387 or TrkC knockdown cells with or without the introduction of DJ-1. P < 0.05 (*), *t*-test. (B) The Luciferase reporter assay for transcriptional activity of CD133 in control SNU387 or TrkC knockdown cells after transfected with or without DJ-1. Error bar; mean ± SEM. *P* < 0.05, *t*-test. (C) The relative mRNA levels of human ABC transporters in TrkC knockdown cells with or without the introduction of DJ-1. P < 0.05 (*), *t*-test. (D) The relative mRNA levels of embryonic stem cells markers and specific liver CSC markers of HCC in PLC/PRF/5 cells after transfected with DJ-1 or TrkC. P < 0.05 (*), *t*-test. (E) The relative mRNA levels of CD133 in PLC/PRF/5 cells after transfected with DJ-1 or TrkC. P < 0.05 (*), *t*-test. (F) The Luciferase reporter assay for transcriptional activity of CD133 in PLC/PRF/5 cells after transfected with DJ-1 or TrkC. Error bar; mean ± SEM. *P* < 0.05, *t*-test. (G) The relative mRNA levels of human ABC transporters in PLC/PRF/5 cells after transfected with DJ-1 or TrkC. P < 0.05 (*), *t*-test.

**Figure S11. Effect of DJ-1 knockdown in PLC/PRF/5 TrkC-DJ-1 cells.** (A) Identification of knockdown of DJ-1 in PLC/PRF/5 TrkC-DJ-1 cells by immunoblotting analysis. (B) The relative mRNA levels of human embryonic stem (hES) cells markers (Oct4, Nanog, Sox2) and specific CSC markers (CD90, CD117, and CK19) of HCC in PLC/PRF/5 TrkC, TrkC-DJ-1, or TrkC-DJ-1 cells with DJ-1 shRNA. P < 0.05 (*), *t*-test. (C) The relative mRNA levels of human ABC transporters in PLC/PRF/5 TrkC, TrkC-DJ-1, or TrkC-DJ-1 cells with DJ-1 shRNA. P < 0.05 (*), *t*-test.

**Figure S12. Effect of TrkC knockdown in the activation of the EMT program in HCC cells.** Immunofluorescence analysis of indicated mesenchymal (fibronectin, vimentin, and N-cadherin) and epithelial (E-cadherin) markers in SNU387 control or SNU387 TrkC-shRNA cells. The nuclei of cells were stained with DAPI (blue).

**Figure S13. Effect of TrkC or DJ-1 in the induction of EMT program.** (A) The relative mRNA levels of epithelial and mesenchymal markers in control SNU387 or TrkC knockdown cells after transfected with or without DJ-1. Error bar; mean ± SEM. *P* < 0.05, *t*-test. (B) The relative mRNA levels of epithelial and mesenchymal markers in PLC/PRF/5 cells after transfected with DJ-1 or TrkC. Error bar; mean ± SEM. *P* < 0.05, *t*-test. (C) The relative mRNA levels of epithelial and mesenchymal markers in PLC/PRF/5 TrkC, TrkC-DJ-1, or TrkC-DJ-1 cells with DJ-1 shRNA. P < 0.05 (*), *t*-test.

**Figure S14. Effect of TrkC knockdown in the induction of the EMT-TFs in HCC cells.** Immunofluorescence analysis of Snail and Twist-1 in SNU387 control or SNU387 TrkC-shRNA cells. The nuclei of cells were stained with DAPI (blue).

**Figure S15. TrkC induces EMT-TFs expression in HCC patients.** The relative mRNA levels of EMT-TFs (FOXC1, FOXC2, Twist-2, ZEB1, and SIP1) in human HCC patients between high or low TrkC expression. The average expression value of TrkC from the TCGA datasets was determined, rank-ordered, and then divided into two equal groups. P < 0.05 relative to TrkC-Low; *t*-test.

**Figure S16. Contribution of TrkC in the EMT activation of HCC patients.** Identification of the correlation between TrkC and expression of EMT-TFs markers (Snail, Twist-1, FOXC1, FOXC2, ZEB1, SIP-1, and Twist-2) in HCC patients. P < 0.0001; spearman or Pearson's correlation coefficient.

**Figure S17. Effect of DJ-1 in the induction of EMT-TFs by TrkC.** The relative mRNA levels of expression of EMT-TFs markers (FOXC1, FOXC2, Snail, SIP-1, Slug, Twist-1, Goosecoid, and Twist-2) in PLC/PRF/5 TrkC, TrkC-DJ-1, or TrkC-DJ-1 cells with DJ-1 shRNA. P < 0.05 (*), *t*-test.
