# Supplementary figures and images for: TrkC-mediated inhibition of DJ-1 degradation is essential for direct regulation of pathogenesis of hepatocellular carcinoma
Source: Cell Death Dis. 2022 Oct 6;13(10):850. doi: 10.1038/s41419-022-05298-3 (PMC9537181; doi:10.1038/s41419-022-05298-3)

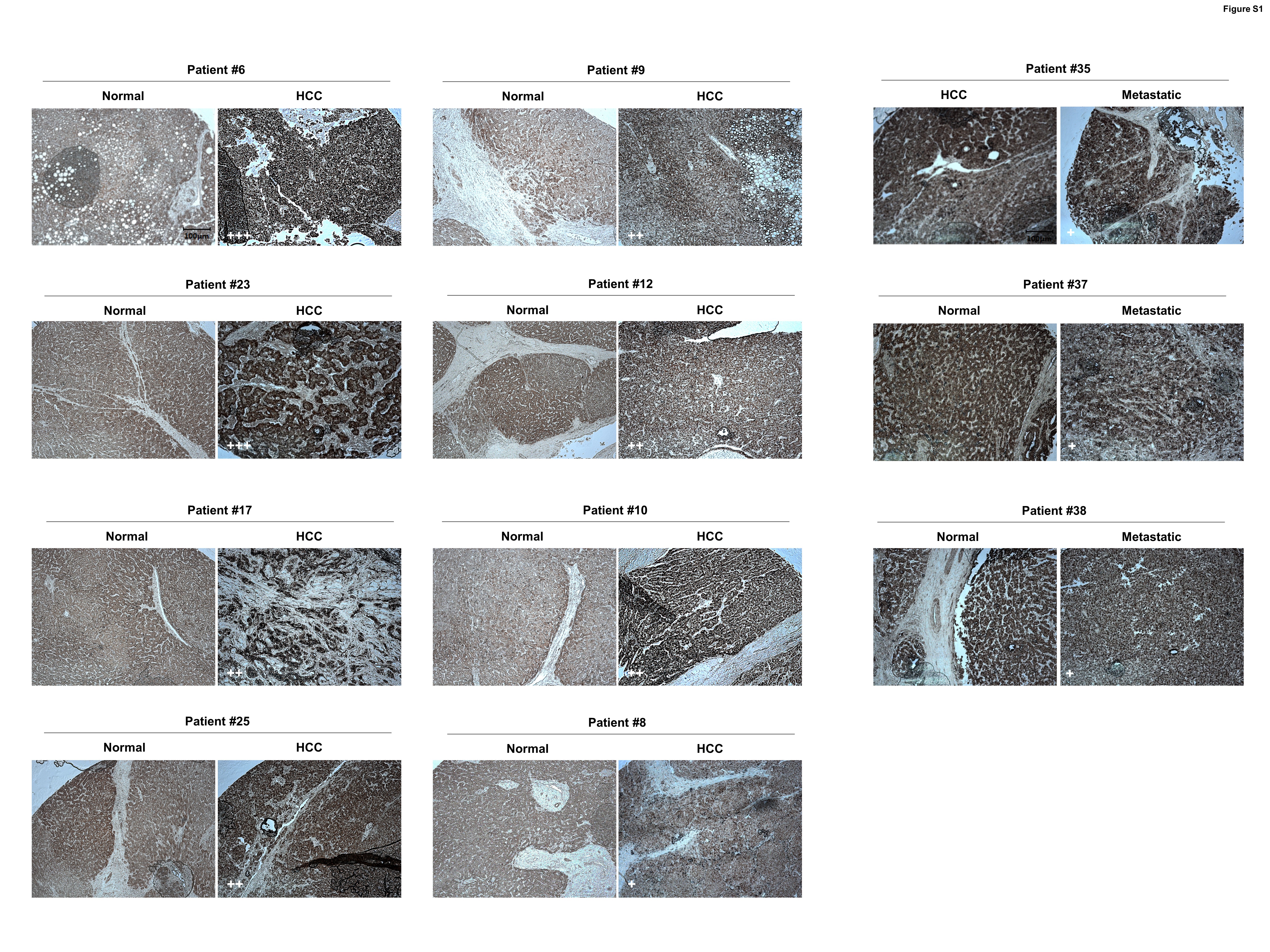

Supplement: Supplementary file 4 — Supplementary Figure 1 [file 41419_2022_5298_MOESM4_ESM.jpg]

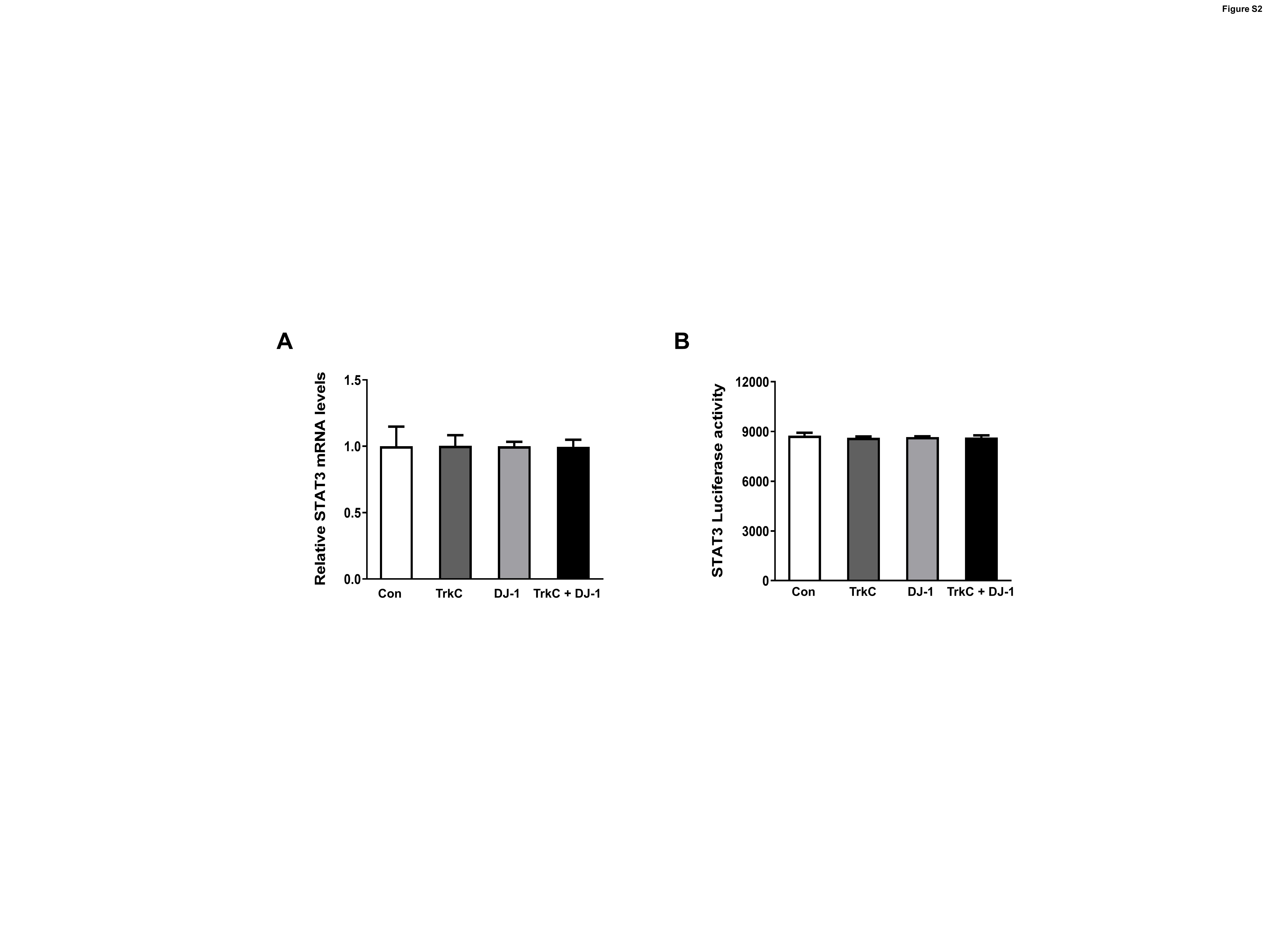

Supplement: Supplementary file 5 — Supplementary Figure 2 [file 41419_2022_5298_MOESM5_ESM.jpg]

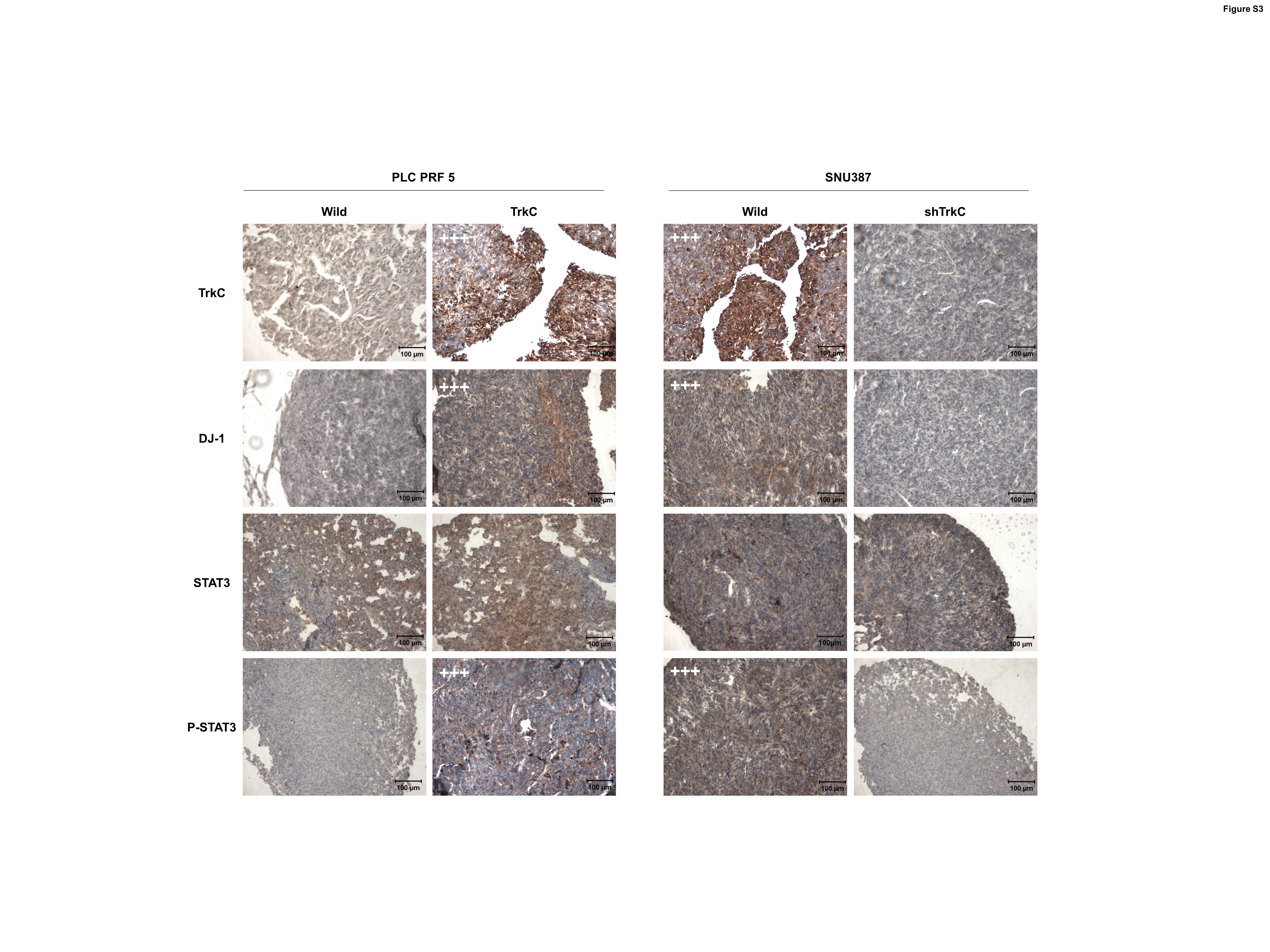

Supplement: Supplementary file 6 — Supplementary Figure 3 [file 41419_2022_5298_MOESM6_ESM.jpg]

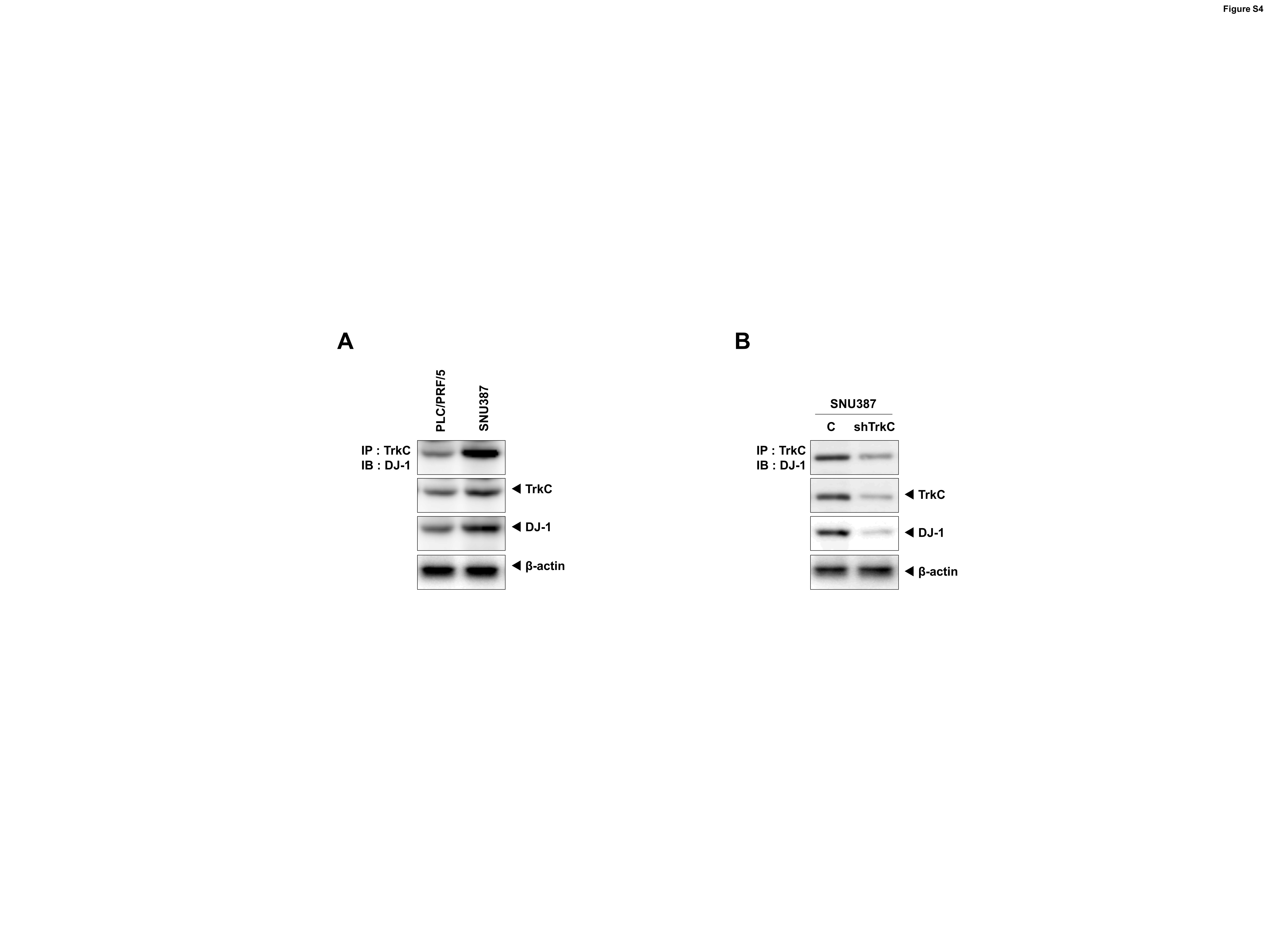

Supplement: Supplementary file 7 — Supplementary Figure 4 [file 41419_2022_5298_MOESM7_ESM.jpg]

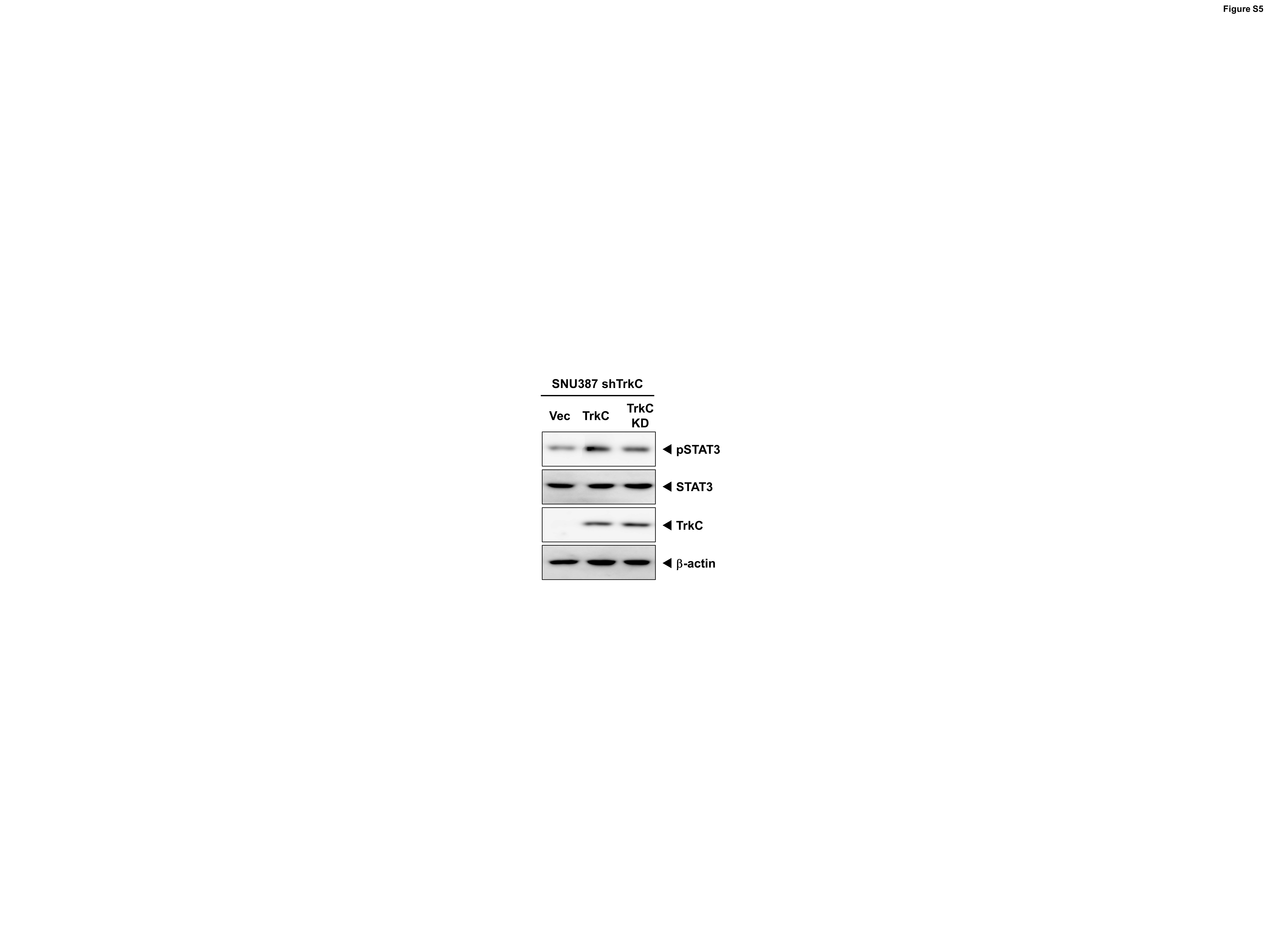

Supplement: Supplementary file 8 — Supplementary Figure 5 [file 41419_2022_5298_MOESM8_ESM.jpg]

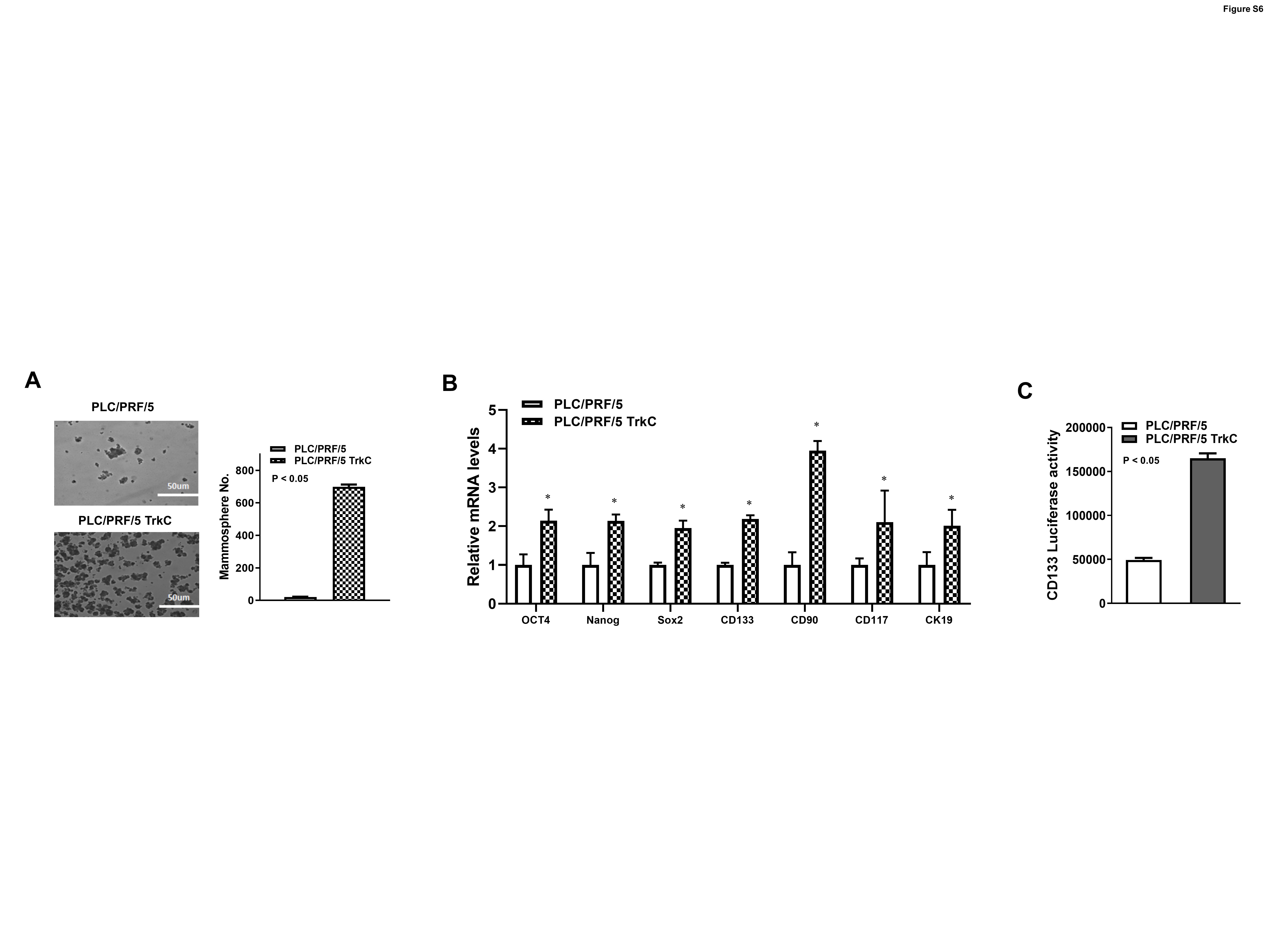

Supplement: Supplementary file 9 — Supplementary Figure 6 [file 41419_2022_5298_MOESM9_ESM.jpg]

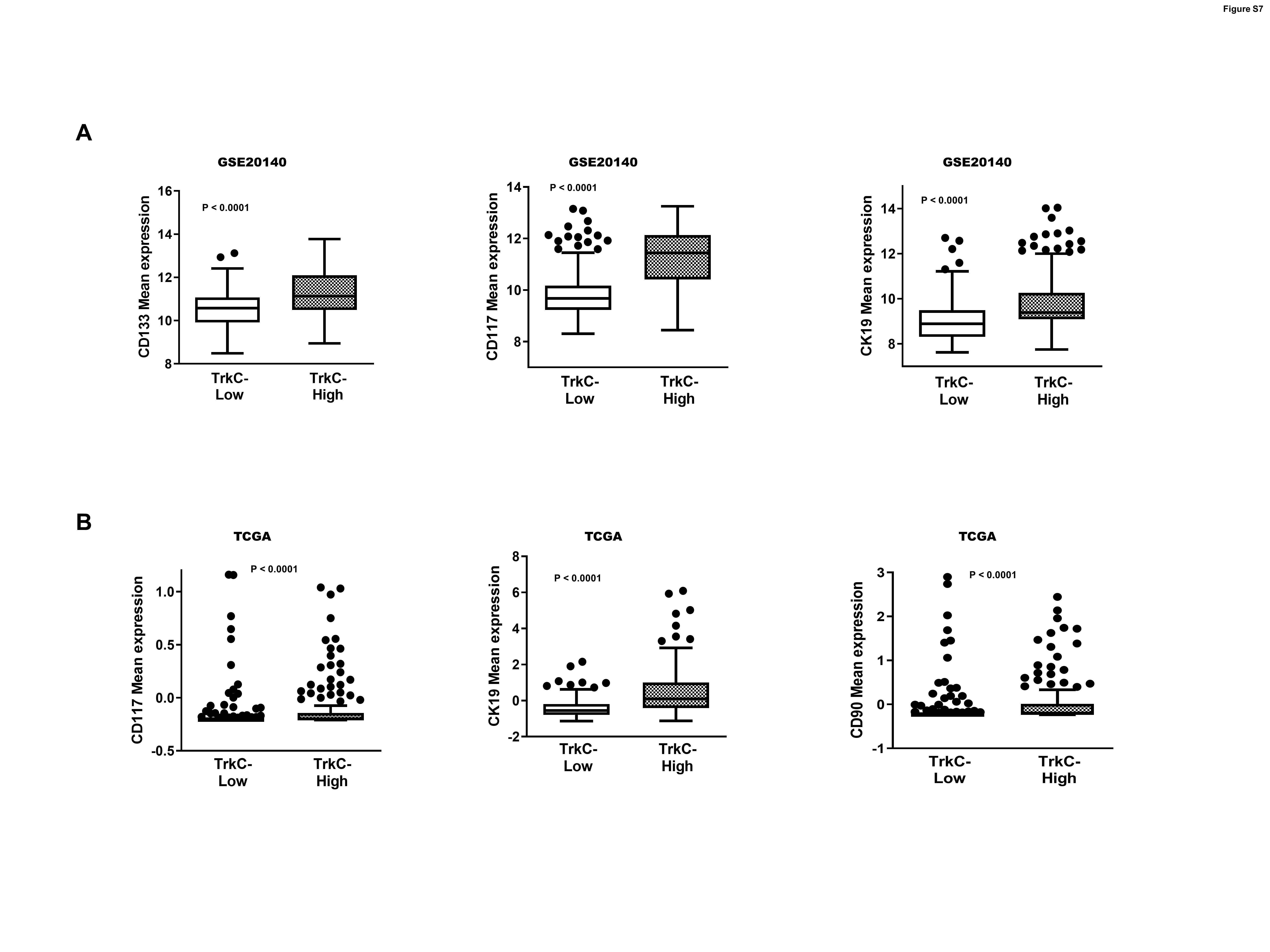

Supplement: Supplementary file 10 — Supplementary Figure 7 [file 41419_2022_5298_MOESM10_ESM.jpg]

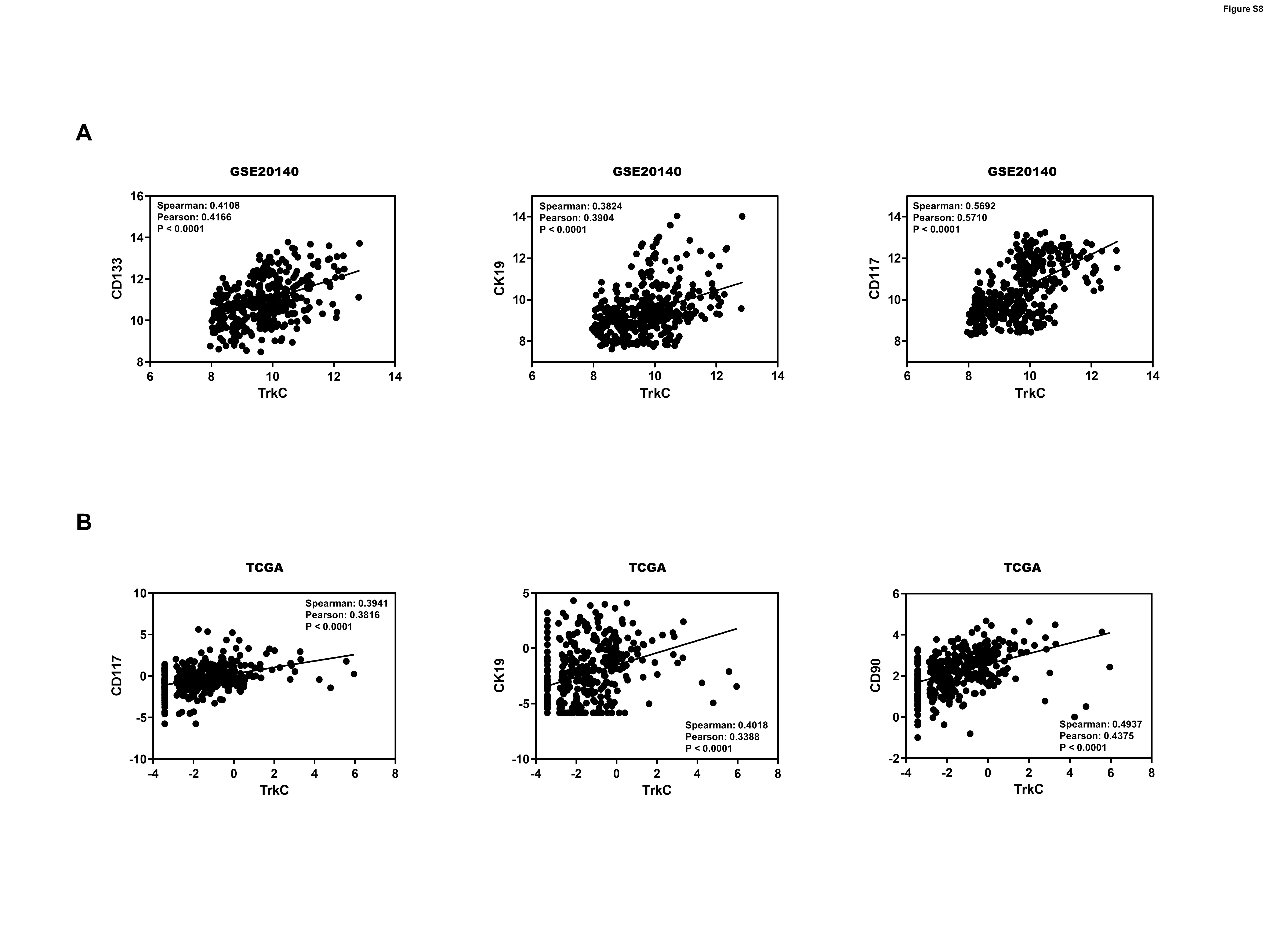

Supplement: Supplementary file 11 — Supplementary Figure 8 [file 41419_2022_5298_MOESM11_ESM.jpg]

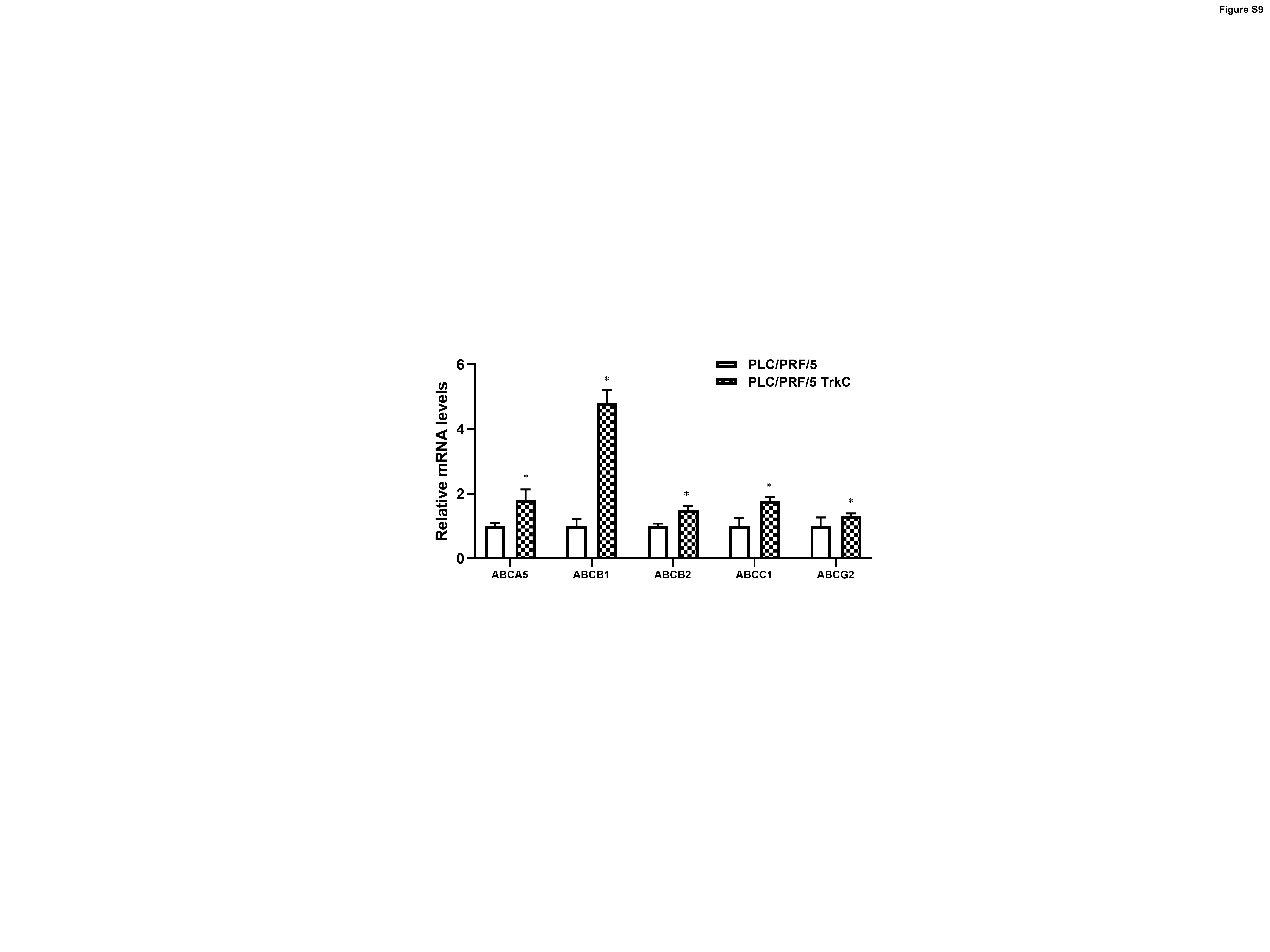

Supplement: Supplementary file 12 — Supplementary Figure 9 [file 41419_2022_5298_MOESM12_ESM.jpg]

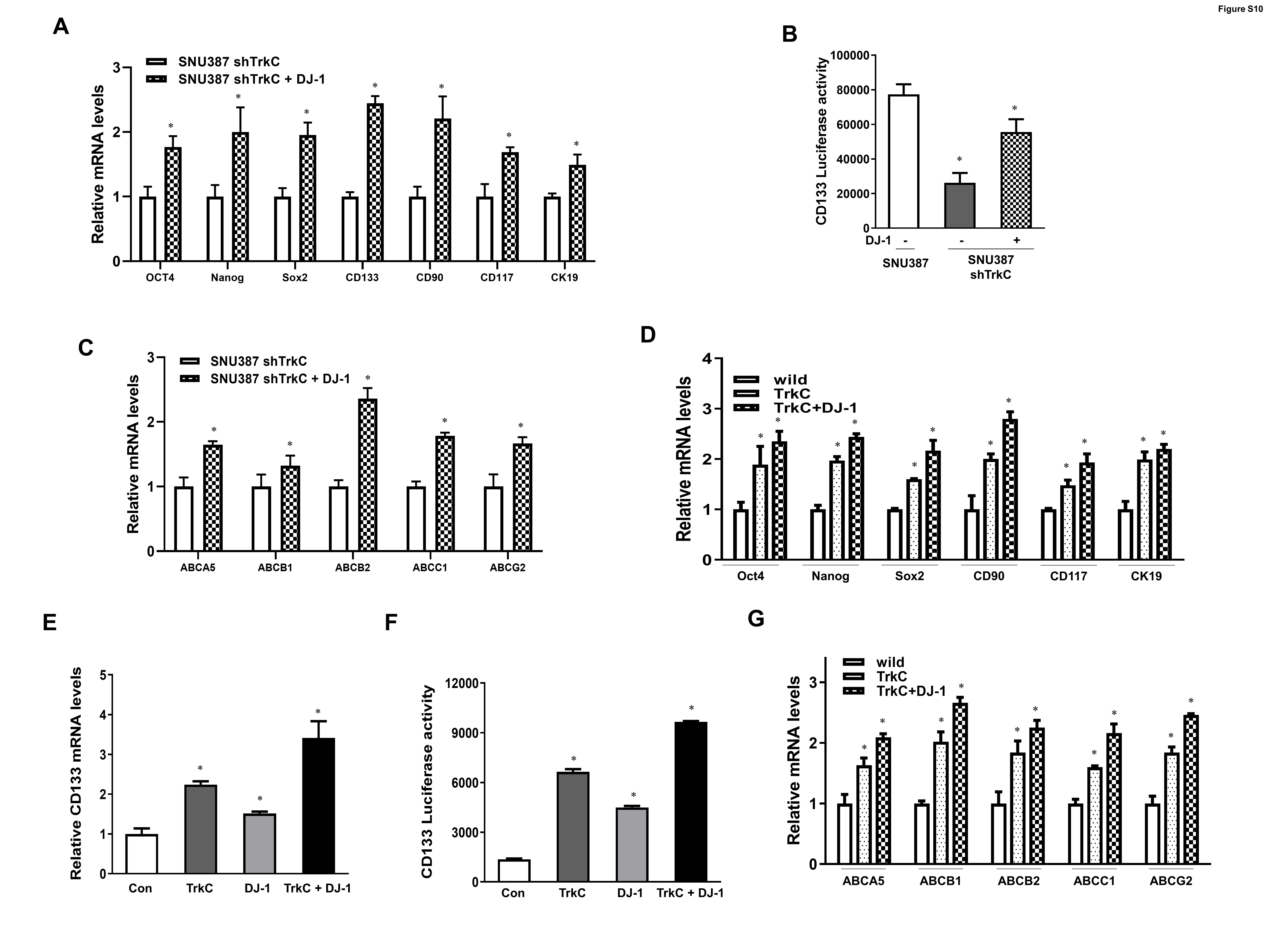

Supplement: Supplementary file 13 — Supplementary Figure 10 [file 41419_2022_5298_MOESM13_ESM.jpg]

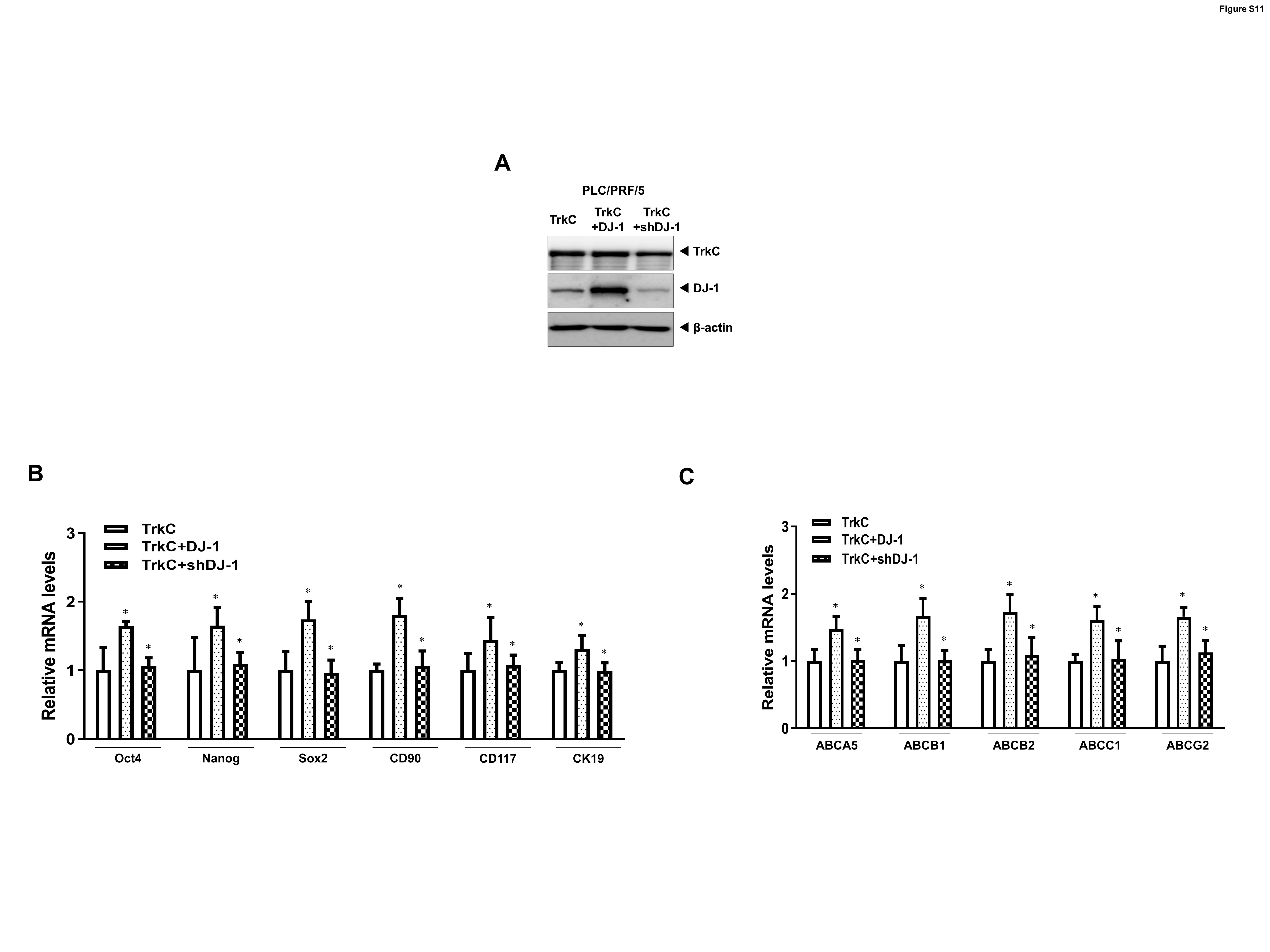

Supplement: Supplementary file 14 — Supplementary Figure 11 [file 41419_2022_5298_MOESM14_ESM.jpg]

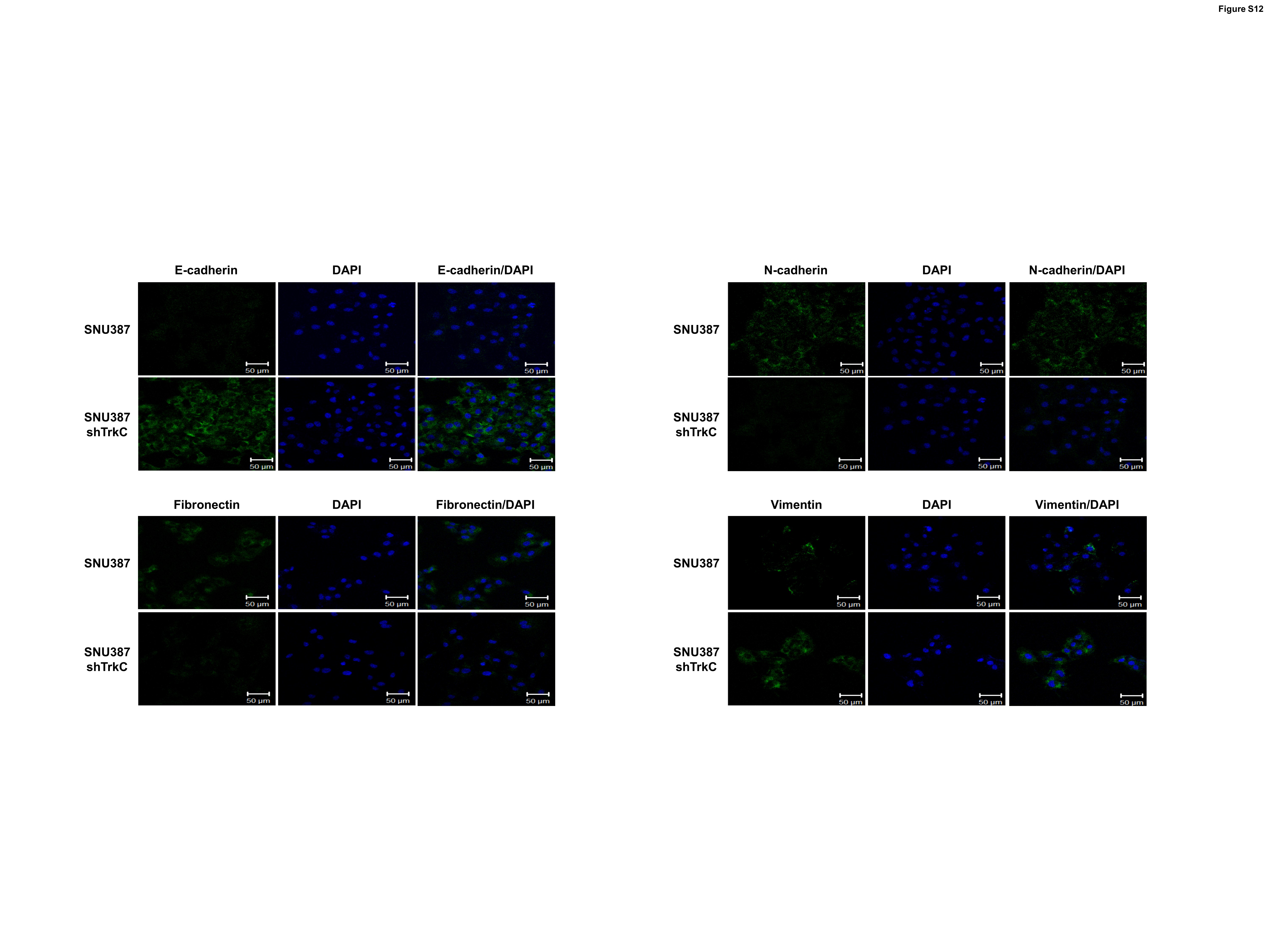

Supplement: Supplementary file 15 — Supplementary Figure 12 [file 41419_2022_5298_MOESM15_ESM.jpg]

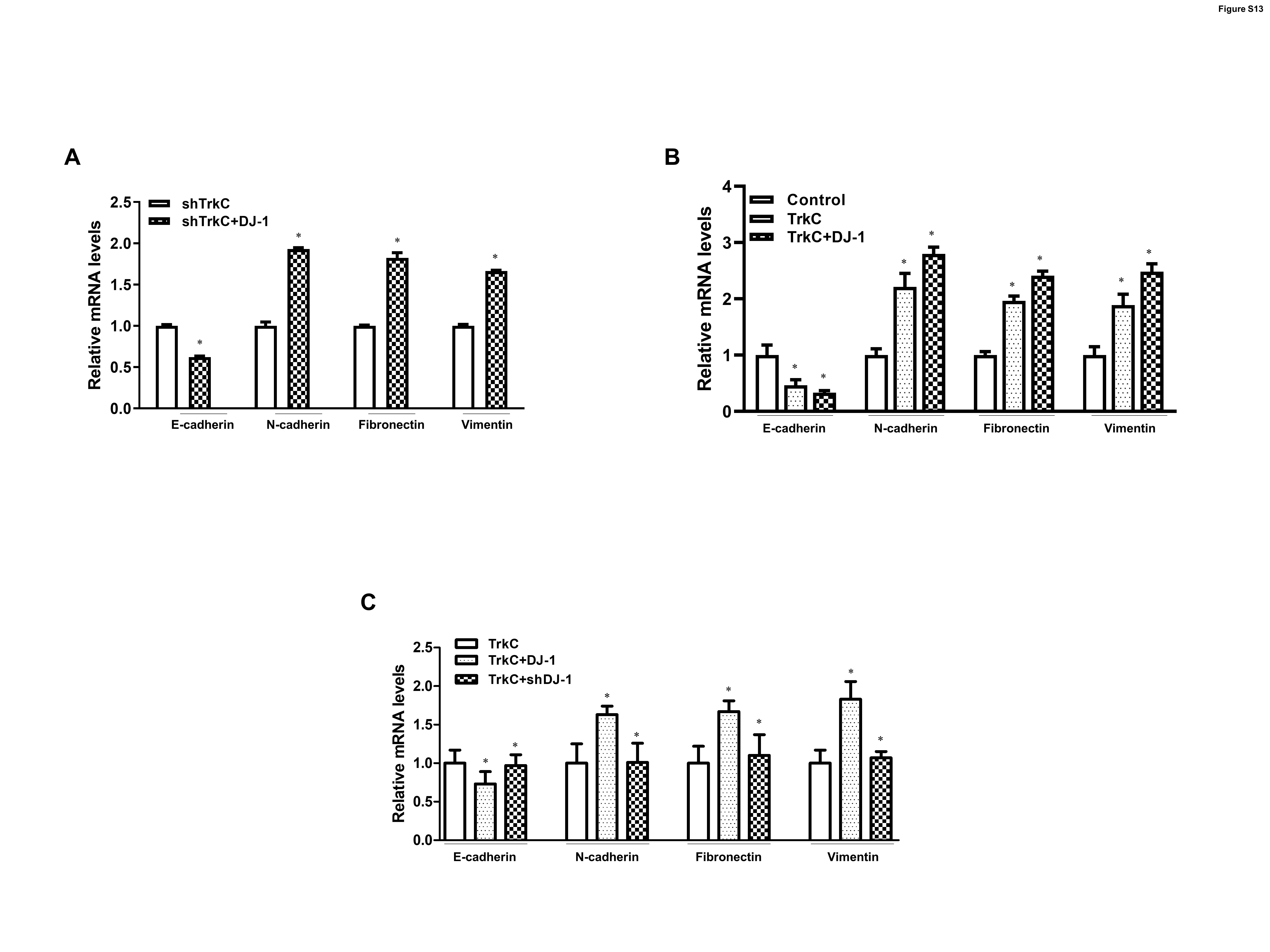

Supplement: Supplementary file 16 — Supplementary Figure 13 [file 41419_2022_5298_MOESM16_ESM.jpg]

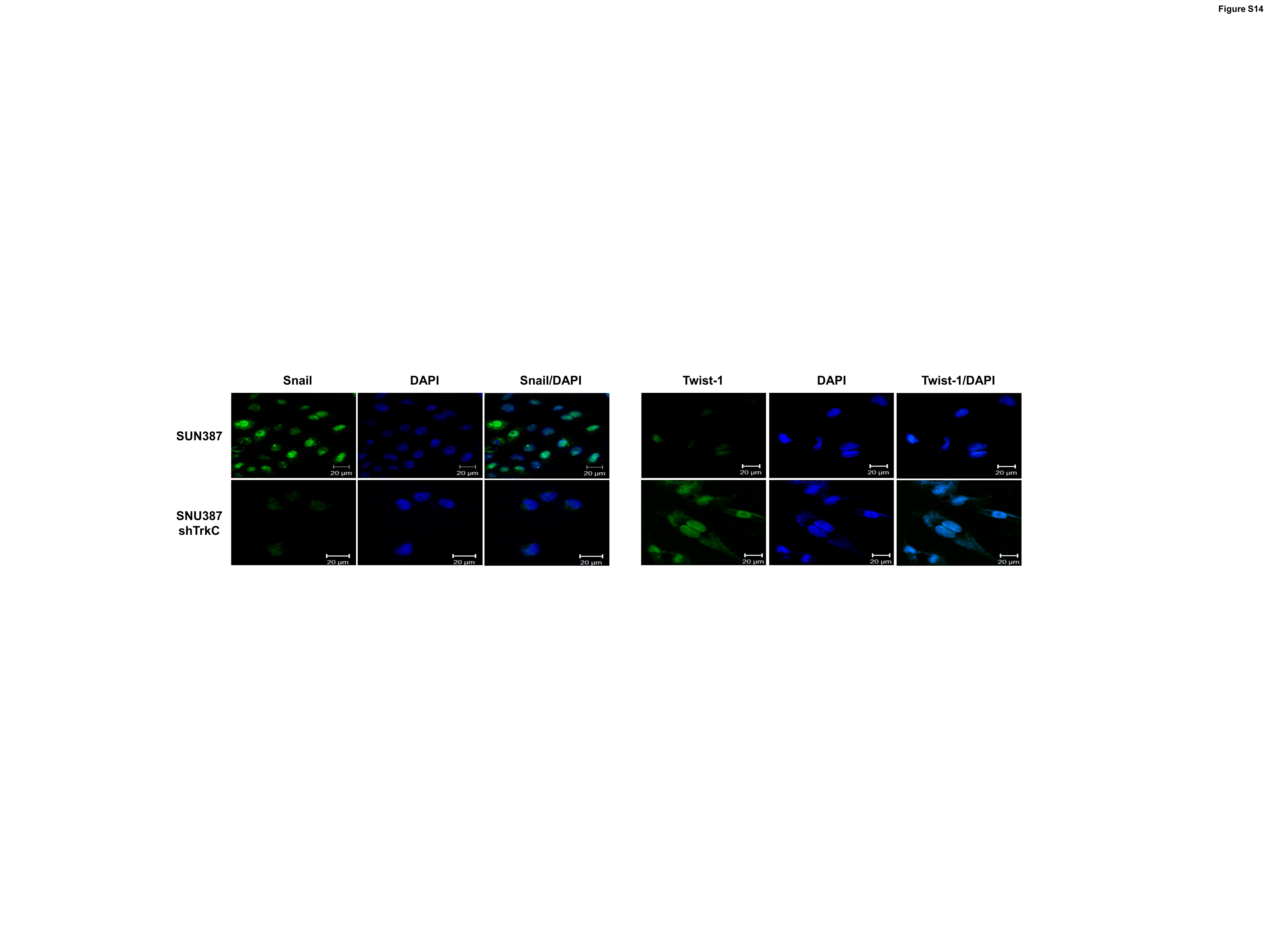

Supplement: Supplementary file 17 — Supplementary Figure 14 [file 41419_2022_5298_MOESM17_ESM.jpg]

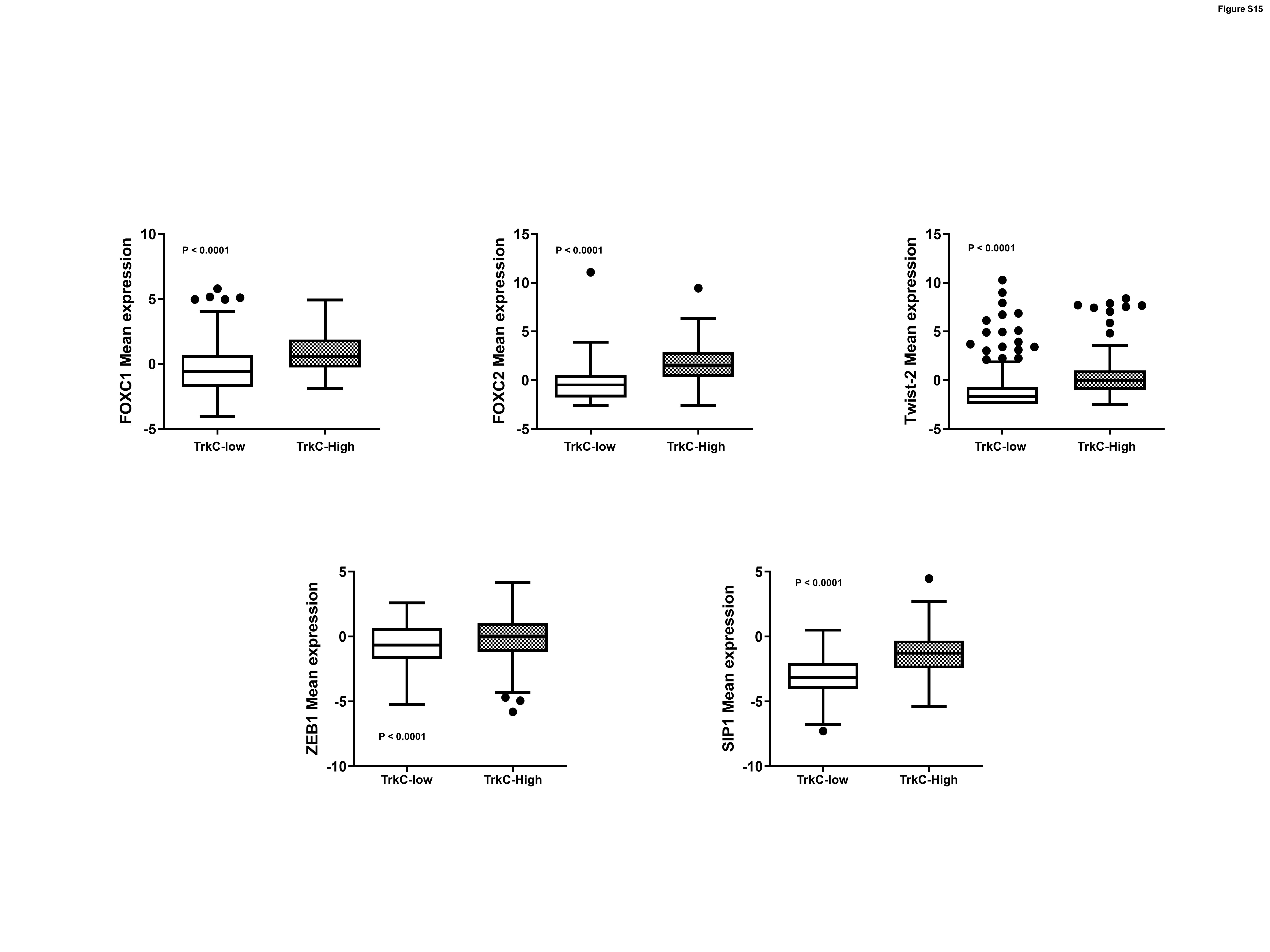

Supplement: Supplementary file 18 — Supplementary Figure 15 [file 41419_2022_5298_MOESM18_ESM.jpg]

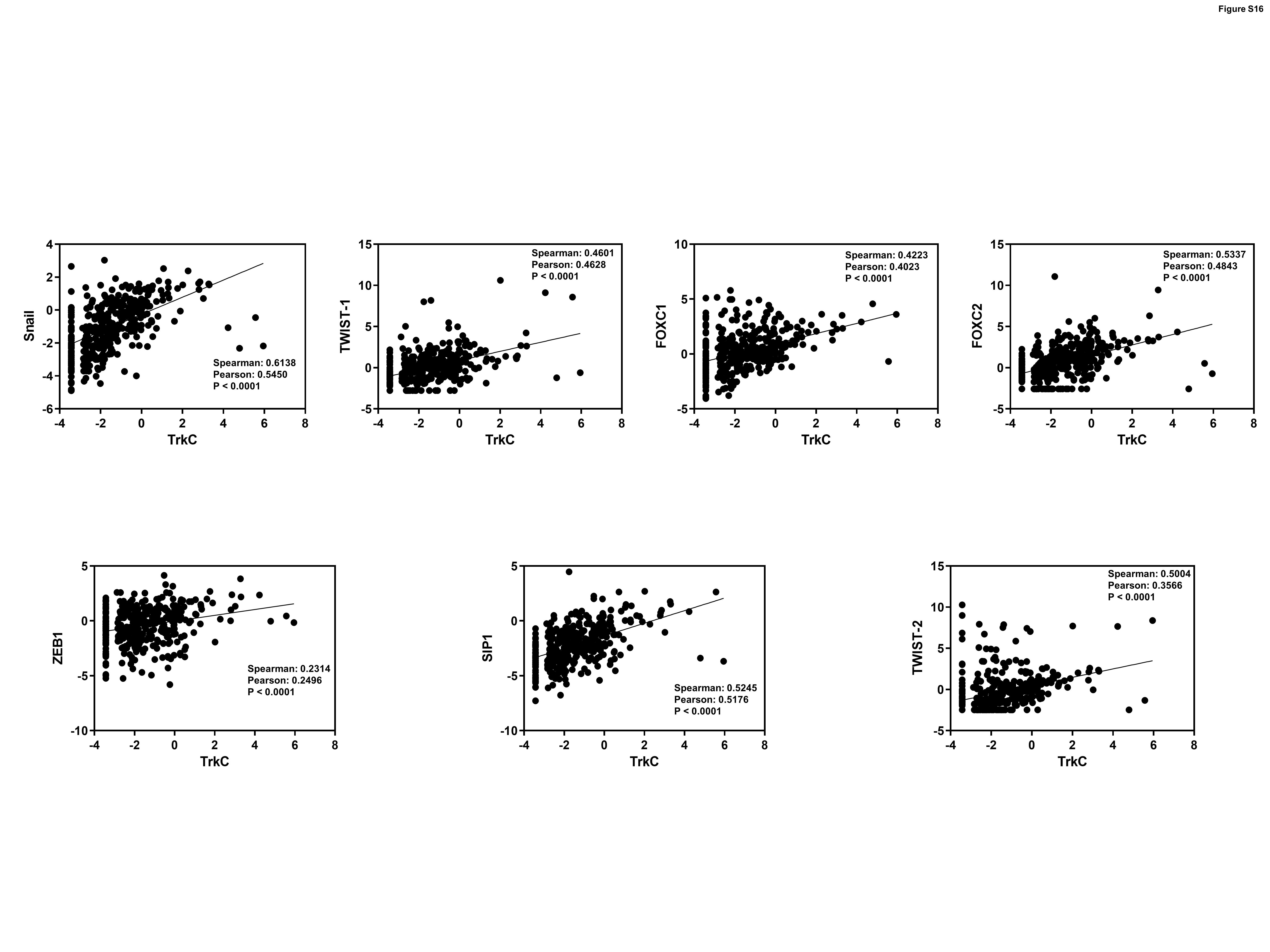

Supplement: Supplementary file 19 — Supplementary Figure 16 [file 41419_2022_5298_MOESM19_ESM.jpg]

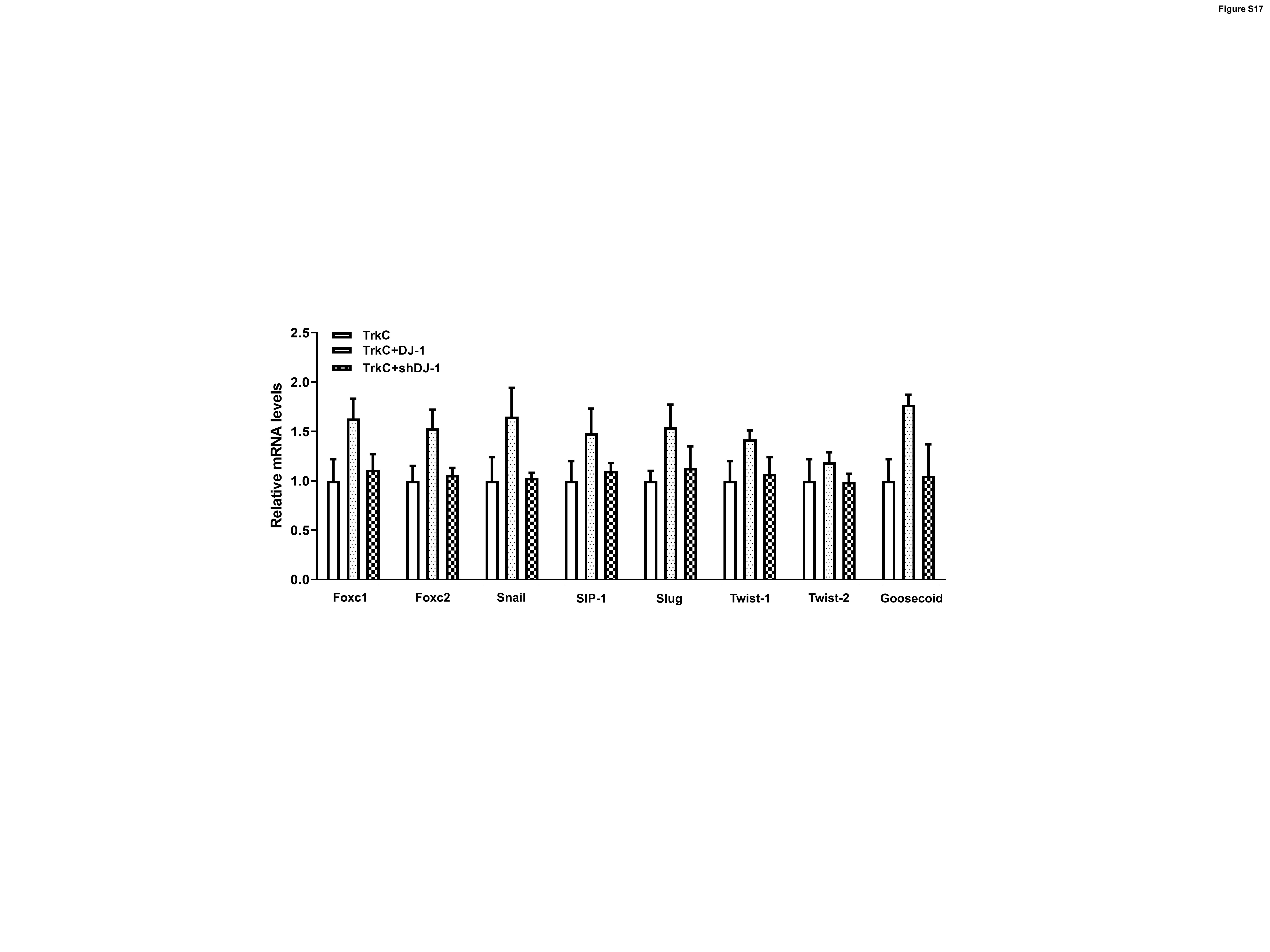

Supplement: Supplementary file 20 — Supplementary Figure 17 [file 41419_2022_5298_MOESM20_ESM.jpg]

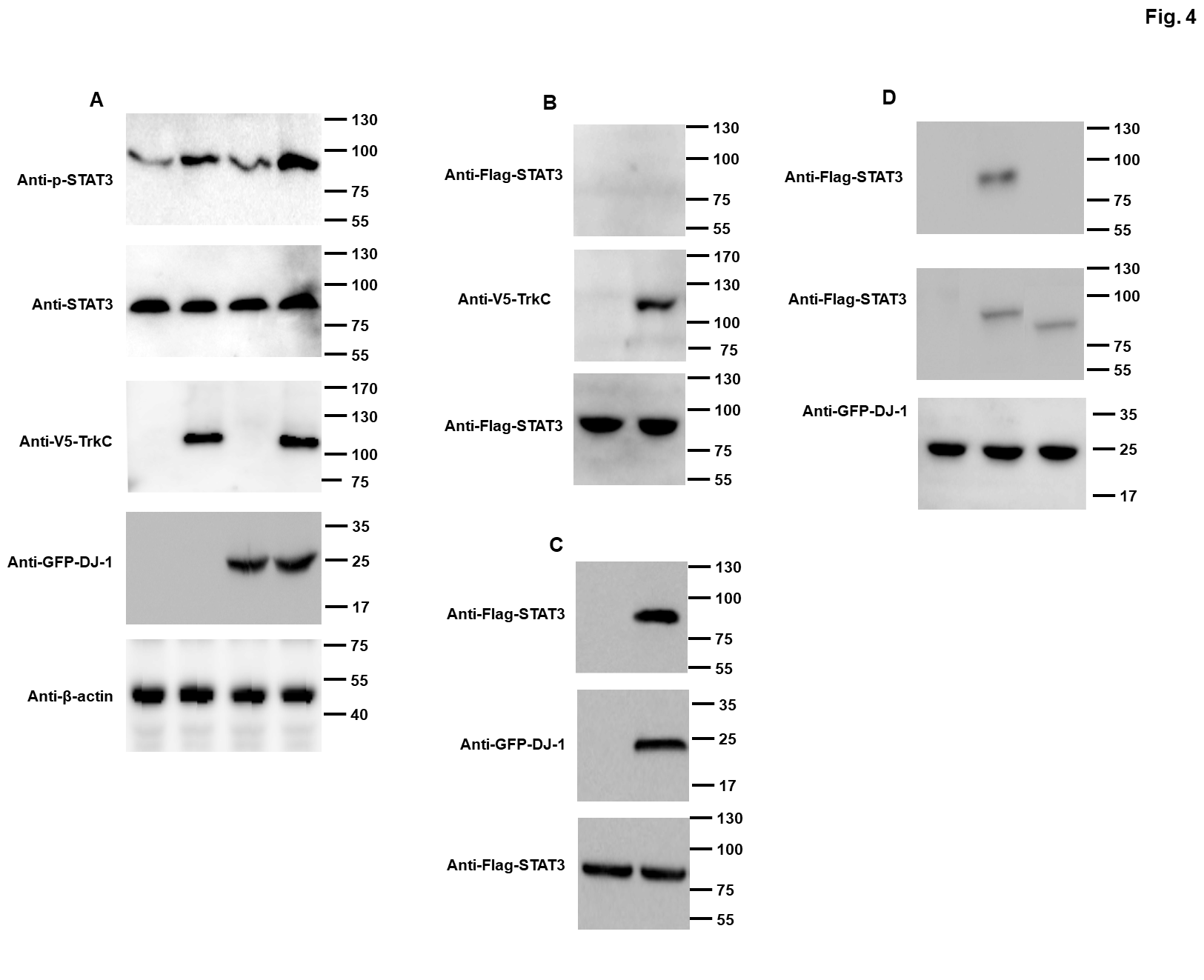

Supplement: Supplementary file 21 — Wester blotting #1 [file 41419_2022_5298_MOESM21_ESM.tif]

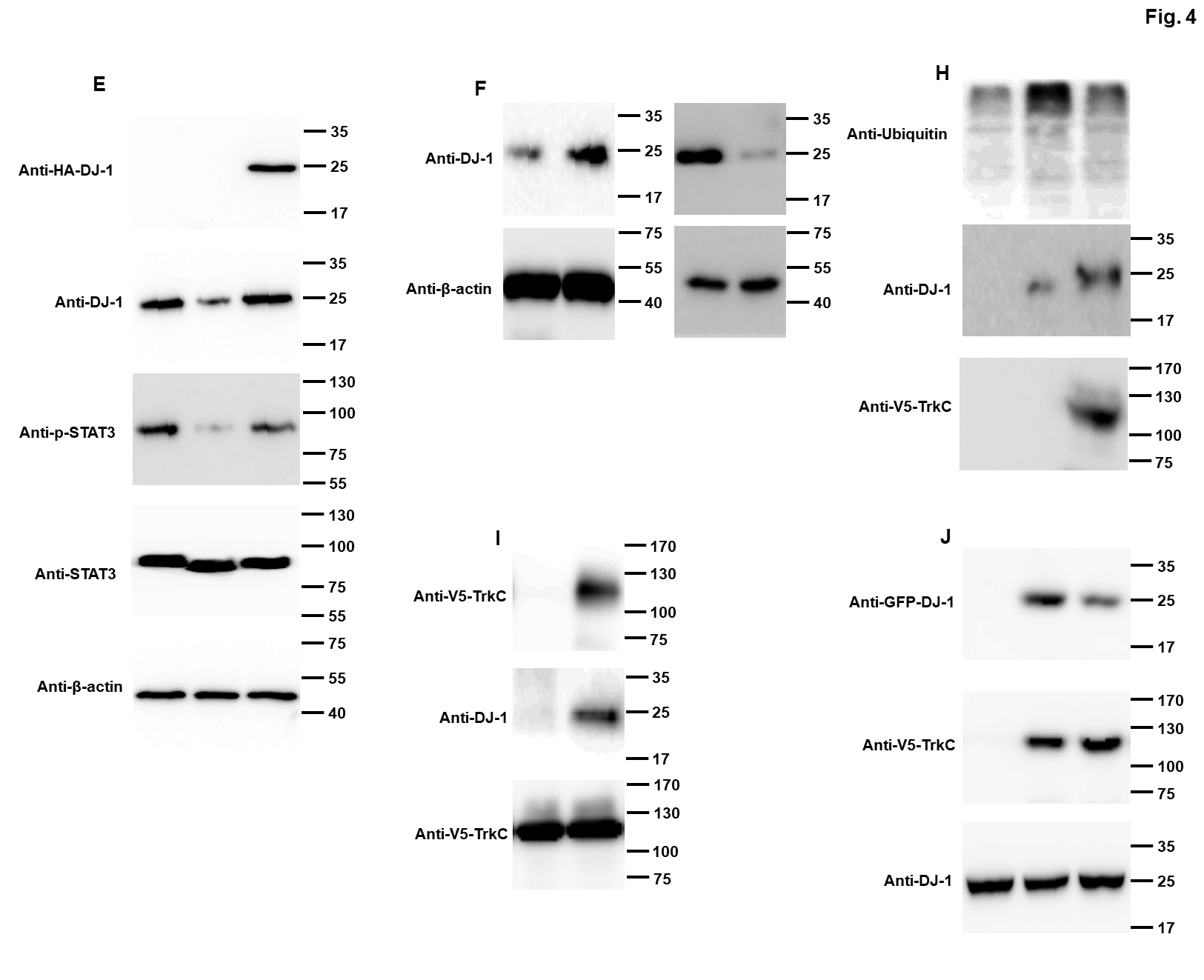

Supplement: Supplementary file 22 — Wester blotting #2 [file 41419_2022_5298_MOESM22_ESM.tif]

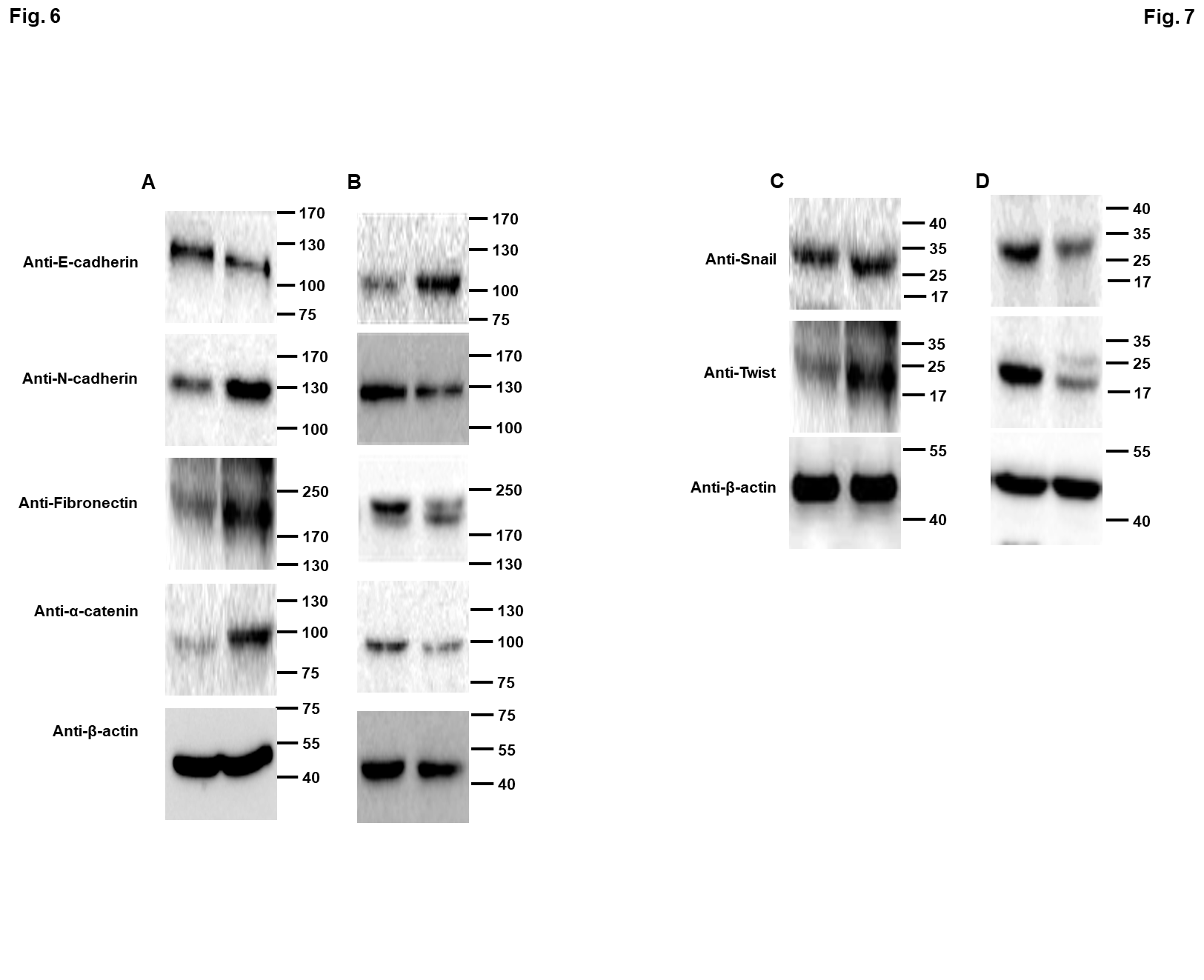

Supplement: Supplementary file 23 — Wester blotting #3 [file 41419_2022_5298_MOESM23_ESM.tif]

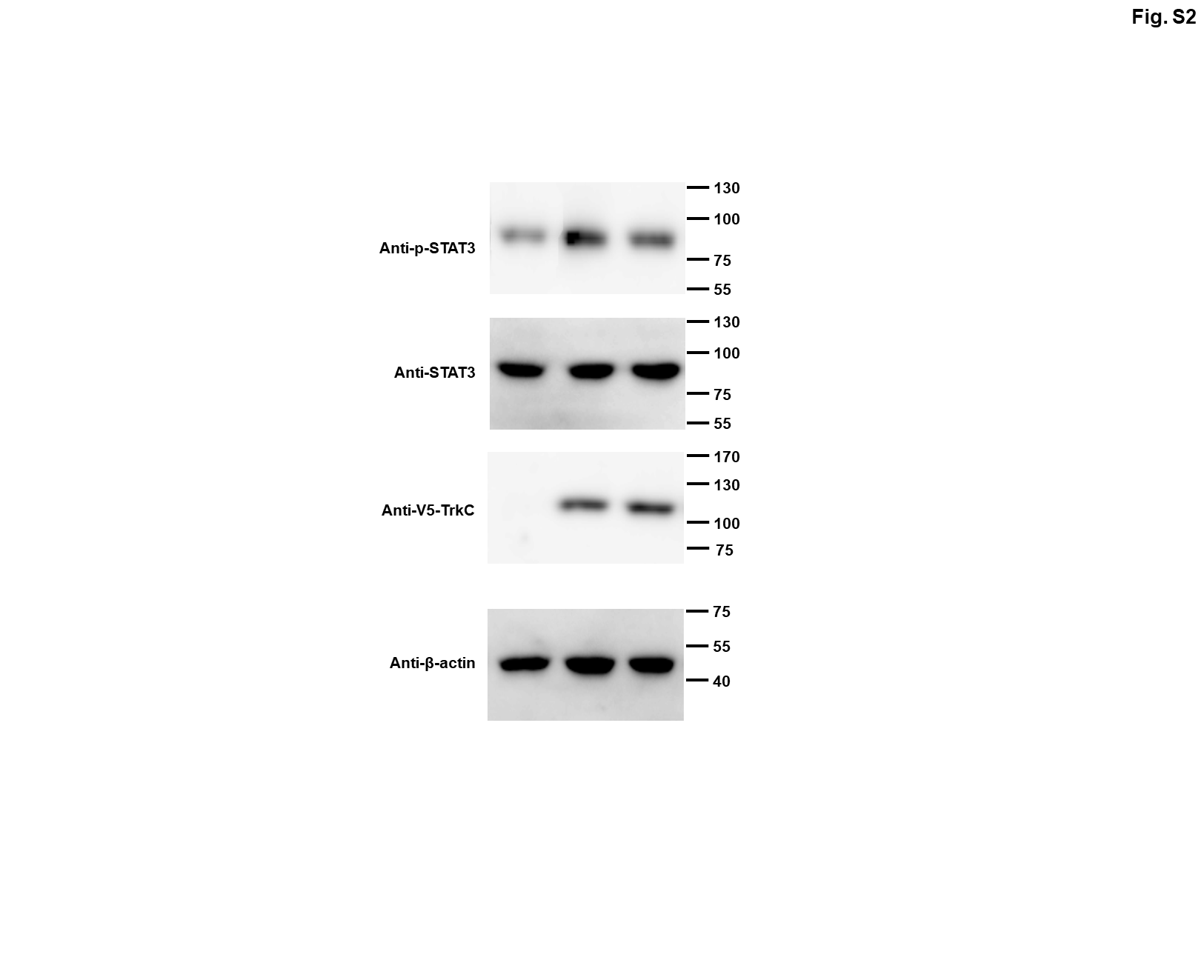

Supplement: Supplementary file 24 — Wester blotting #4 [file 41419_2022_5298_MOESM24_ESM.tif]
